# Associations between the Chinese physical education and health Curriculum model to the psychological stress of junior middle school students: The chain mediating role of psychological resilience and physical self-efficacy

**DOI:** 10.1371/journal.pone.0360804

**Published:** 2026-10-26

**Authors:** Lin Shi, Liping Liu, Yanhao Zong, Hu Lou, Aimin Ma

**Affiliations:** Nantong University, Nantong, Jiangsu Province, China; Rehabilitative Hospital Affiliated to Fujian University of Traditional Chinese Medicine: Fujian University of Traditional Chinese Medicine Affiliated Rehabilitative Hospital, CHINA

## Abstract

To explore the correlational relationship between the Chinese physical education and health curriculum model (CPEH Curriculum) and psychological stress among junior middle school students, as well as the statistical mediating roles of psychological resilience and physical self‑efficacy within such associations.A total of 395 junior middle school students were investigated using four standardized measurement scales. Correlation analysis indicated clear associations among the main variables.Psychological resilience and physical self-efficacy were negatively associated with students’psychological stress, whereas the CPEH Curriculum showed a positive direct correlational linkage with psychological stress. while positive correlations existed between the curriculum model and the two psychological variables. Chain mediation analysis revealed four statistical correlational pathways. the direct statistical correlational linkage related to the curriculum model, the independent mediating effect of psychological resilience, the independent mediating effect of physical self-efficacy, and the chain mediating effect of the two variables. This study indicates that the CPEH Curriculum is correlated with students’ psychological stress through the chain mediation of psychological resilience and physical self-efficacy based on cross-sectional evidence.No causal inferences can be drawn from the present single-time-point dataset.

## Introduction

The prevalence of high psychological stress among Chinese junior middle school students constitutes a prominent social concern [1]. It is acknowledged that moderate stress is not entirely negative, as it may foster learning motivation and potential [2]. However, excessive stress produces multifaceted adverse outcomes, encompassing mental and physical health issues such as anxiety, depression, and sleep disorders [3,4]. Furthermore, it precipitates interpersonal difficulties, including weakened social skills and disharmonious parent-child relationships [5,6], alongside diminished academic performance and engagement [7]. Hence, research into the correlational mechanisms of this stress is vital for formulating effective strategies to safeguard student well-being.The psychological regulation theory provides a foundational perspective, positing that physical activity serves as an effective regulator of psychological state [8,9]. Engaging in such activity facilitates cognitive diversion from stressors, leading to higher emotional well-being and more positive mental health. Furthermore, the social dimensions of physical activity—such as building interpersonal relationships, strengthening personal sense of belonging, and sharing experiences—further contribute to lower levels of stress [10].Complementing this, self-efficacy theory highlights a positive correlation between students’ self-belief and their athletic performance [11]. Those with higher self-efficacy typically set more ambitious goals and, through persistent effort, achieve success and continual improvement. Bandura's social cognitive theory (1986) elaborates on this by framing human functioning as a dynamic interaction of personal, behavioral, and environmental factors [12]. Within this framework, sports activities constitute a rich social context where cooperation, competition, and shared objectives enhance social bonds, foster belonging, and mitigate feelings of loneliness. The self-efficacy enhancement theory further suggests that physical activity links to higher a sense of mastery and competence, which are crucial for coping with psychological stress [13].A significant gap in the literature concerns the interplay between the psychological regulation and self-efficacy enhancement mechanisms. It remains unclear whether these pathways operate sequentially or concurrently in stress adaptation [14,15].

The majority of existing research has focused on voluntary, leisure-time physical activity, leaving a notable scarcity of studies on the role of structured, curriculum-based physical education (PE) [16]. This gap is particularly relevant in the contemporary Chinese educational context. The implementation of the “Double Reduction” policy has significantly lengthened the school day, often exceeding 12 hours for junior middle school students, thereby reducing opportunities for independent physical activity [16]. Paradoxically, this policy shift has been coupled with a heightened emphasis on PE. The 2022 compulsory education curriculum plan increased the proportion of PE and health courses to 10%−11% of the total curriculum, establishing it as a core subject. The accompanying Physical Education and Health Curriculum Standards (2022 Edition)mandates a minimum of three weekly class hours for junior middle schools, advocating an instructional philosophy of “mastery, diligent practice, and regular competition” [16]. This policy landscape underscores the critical importance of exploring the associations between structured PE and student well-being.

The CPEH Curriculum is a systematic framework designed to promote students’ holistic development through structured physical activity [17]. Its core principles include: (1) ensuring an appropriate exercise load per session (with a target intensity of 125–160 bpm and density exceeding 75%); (2) incorporating approximately 10 minutes of dedicated physical fitness practice; and (3) teaching sports skills through engaging activities and competitions [18]. While no study has directly investigated the relationship between the CPEH Curriculum and psychological stress among junior middle school students in China, extant literature suggests that adolescent physical activity correlates with coping with developmental and academic pressures [19]. Research on the CPEH Curriculum confirms its efficacy in higher physical fitness, stimulating motivation and interest, and greater self-confidence and academic performance in PE [20]. Crucially, maintaining an average heart rate within the 140–160 bpm range has been shown to correlate with milder logical stress responses and lower logical discomfort [21]. The collaborative nature of the CPEH Curriculum also inherently promotes social interaction,teamwork,andresilience. Therefore, we hypothesize:H1:The CPEH curriculum model is hypothesized to correlate with psychological stress among junior middle school students.

Psychological resilience—defined as the capacity to adapt successfully to adversity—is a critical protective factor during adolescence, a period marked by significant change and stressor exposure [22–24]. Empirical evidence consistently demonstrates that, purposeful physical activity significantly correlates with more favorable mental health outcomes including resilience [25,26]. Regular participation not only correlates with more positive emotional states but also higher self-confidence and coping skills, thereby fostering greater resilience [27,28]. Accordingly, it is proposed that the model correlates with students’ ability to withstand stress by alongside elevated this key psychological resource. We thus hypothesize:H2:Psychological resilience mediates the relationship between the CPEH Curriculum and psychological stress.

Physical self-efficacy, an individual's confidence in their physical capabilities, is a potent predictor of sustained participation in physical activity [29]. Although its direct link with stress in this demographic warrants further investigation, general self-efficacy is known to correlate with mental health, earning motivation, and the adoption of adaptive coping strategies, which can mitigate stress [30]. The transferable nature of self-efficacy implies that confidence gained in physical domains can generalize to other life situations [31]. The structured skill development, fitness training, and competitive elements of the Chinese model are posited to significantly correlate with students’ physical self-concept and self-efficacy [32]. This leads to the following hypotheses:H3:Physical self-efficacy is hypothesized to correlate negatively with psychological stress among junior middle school students.H4:Physical self-efficacy mediates the relationship between the CPEH Curriculum and psychological stress.

A robust positive correlation exists between psychological resilience and self-efficacy [33]. Individuals with higher self-efficacy generally demonstrate greater resilience and more effective adjustment strategies. Self-efficacy also plays a key role in emotional regulation, influencing learning and adaptation by affecting motivation and attitude [34]. This suggests a synergistic relationship where the CPEH Curriculum may correlate with resilience, which in turn correlates with higher self-efficacy, forming a cascading protective association with stress.Therefore, we propose a chain-mediation model:H5:Psychological resilience and physical self-efficacy sequentially mediate the relationship between the CPEH Curriculum and psychological stress.

In summary, a conceptual framework links the CPEH Curriculum, psychological resilience, physical self-efficacy, and psychological stress. While prior research offers partial support for these associations, the underlying mechanisms, particularly the proposed chain-mediation effect, remain empirically unexamined. This study aims to address this gap by investigating the correlational and predictive relationships between the CPEH Curriculum and psychological stress, with a specific focus on the chain-mediating roles of psychological resilience and physical self-efficacy. The comprehensive hypothetical model is presented in Fig 1.

**Fig 1:**
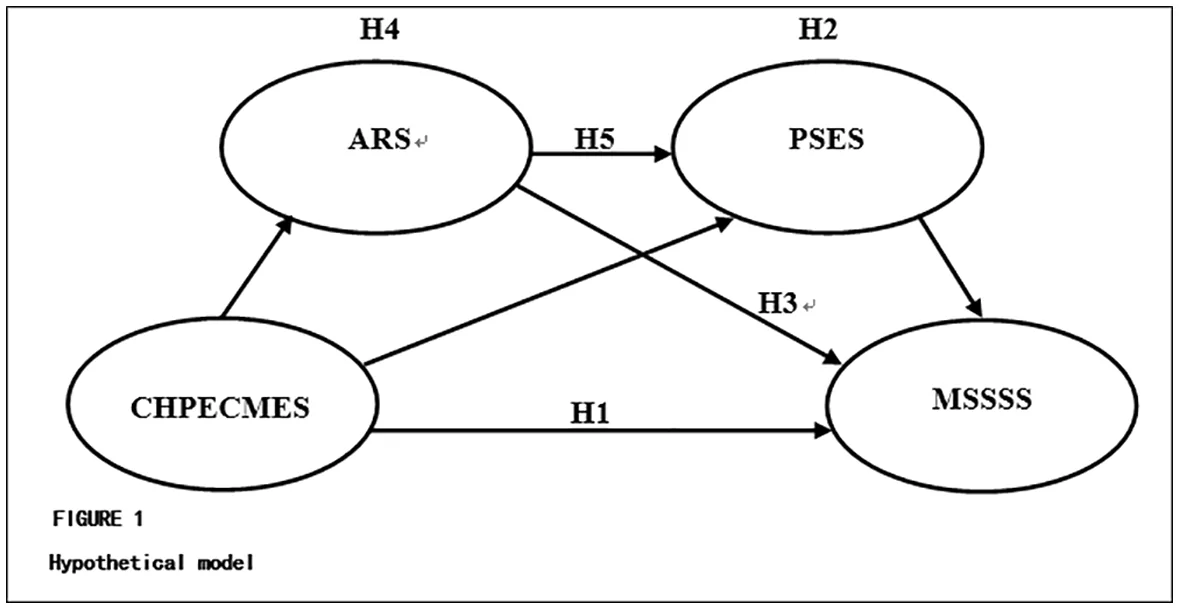
Hypothesized statistical chain-mediation model among the CPEH curriculum model, psychological resilience, physical self-efficacy and psychological stress among junior middle school students. All path coefficients displayed in this model are standardized estimates. CPEH Curriculum = Evaluation score of the CPEH Curriculum; ARS = Adolescent Resilience Scale; PSES = Physical Self-Efficacy Scale; MSSSS = Middle School Student Stress Scale.

## Methods

### Study design

We contacted the participating schools in advance before distributing questionnaires. Questionnaires were handed out uniformly and collected on the spot. After removing invalid questionnaires with excessive missing items or regular repetitive answers, valid data were sorted out for subsequent statistical analyses

Data collection was completed uniformly in November 2024. Each participant gave their informed consent prior to participation, and signed consent forms were collected from all subjects voluntarily. The research background and objectives were fully stated in the consent documents. All data were processed anonymously throughout the study. No physical harm would be caused to participants, and no sensitive personal information or commercial interests were involved. Ethical approval was obtained from the Ethics Committee of Nantong University (approval No. 2022(70)). This study was conducted in full compliance with the ethical principles outlined in the Declaration of Helsinki

This study adopts a cross-sectional design with single-time-point data collection, which can only verify correlational and predictive relationships rather than strict causal relationships between variables. For all minor students, written consent from parents was obtained.

### Sample

Questionnaires were distributed to all junior high school students from nine classes adopting the CPEH Curriculum using convenience sampling. In total, 426 questionnaires were sent out, among which 31 were ruled out as invalid. Finally, 395 valid questionnaires were retrieved, corresponding to an effective response rate of 92.72%. The sample consisted of 195 boys and 200 girls, including 140 seventh-grade students, 145 eighth-grade students and 110 ninth-grade students. Participants were aged 13–16 years, with a mean age of (14.14 ± 0.87) years old. Basic socioeconomic information covering parental education and self-rated family economic status was collected as supplementary demographic data of participants.Every participant filled out the questionnaire voluntarily with full understanding of the study.

Convenience sampling was applied in this research, which falls into the category of non-probability sampling. All valid raw data used for analysis are available in S1 Dataset.

### CPEH curriculum implementation

The CPEH curriculum is a structured physical-education model based on China’s 2022 Compulsory Education Physical Education and Health Curriculum Standards, following the philosophy of “mastery, diligent practice, and regular competition”.

The curriculum includes three core components: motor‑skill teaching and practice, in‑class physical‑fitness training, and classroom competitions and games. Its goals are to improve students’ motor competence, physical fitness, sports participation, teamwork and mental well‑being.

Implemented throughout the academic year, the CPEH curriculum provides at least three 45‑minute lessons per week. Each lesson follows a fixed sequence: 5‑min warm‑up, 20‑min skill practice, 10‑min fitness training, 7‑min competitive games, and 3‑min cool‑down and summary. The target heart rate is 140–160 bpm with class exercise density above 75%.

All participating PE teachers hold bachelor’s degrees in physical education and valid teaching certificates. Before implementation, teachers completed mandatory training organized by the local education bureau, covering curriculum interpretation, lesson design, exercise‑load monitoring, competition organization and safety management. School teaching groups conducted regular inspections to guarantee curriculum fidelity.

Different from traditional teacher‑centered PE classes with unstable exercise intensity and few structured competitions, the CPEH curriculum features standardized exercise‑load monitoring, embedded in‑class fitness training, integrated “learning‑practice‑competition” design, and unified criterion‑referenced assessments for motor skills and physical fitness.

### Measuring tools

#### The CPEH curriculum evaluation scale (CPEH Curriculum1).

The scale was compiled by Liu et al. [35], which was mainly based on the three key points of exercise load, physical fitness practice, and sports skills emphasized in the CPEH Curriculum and the theory of sports education [36]to investigate the effect of the CPEH Curriculum in the physical education course. The scale consists of 23 items, including three subscales: teacher behavior, student behavior, and interactive behavior. In this study, the student behavior subscale was used as a measurement tool, including 11 items, which are three items of course content and teaching methods (the degree of structure of course content, the innovation of teaching methods, the rationality of teaching time allocation), five items of exercise load and physical response (exercise load, sports skill mastery, physical development, interest attitude, physical fatigue), and three items of course effect and gain (strengthen physical fitness, tempering will, and improving mental health). The scale adopts the Likert 5-level scoring method. The higher the score, the better the learning effect of junior middle school students in the physical education classroom using the CPEH Curriculum. The Cronbach’s α coefficient of this scale in this study is 0.751.

#### The Stressor Scale for Middle School Students (MSSSS).

The scale was compiled by Zheng [37] et al., using the Likert 5-level scoring method, and was composed of 39 items, including seven subscales: learning stress, teacher stress, family environment stress, parental education style stress, classmate and friend stress, social and cultural stress, and self-physical and mental stress. Higher scores indicate higher levels of psychological stress for middle school students. The Cronbach's α coefficient of the total scale is 0.987.All stress-related outcome variables in the present study correspond to the construct measured by the Middle-School Student Stress Scale.

#### The Resilience Scale for Chinese Adolescents (ARS).

The scale was developed by Hu [38] et al., using the Likert 5-level scoring method. It consisted of 27 items, including five subscales of goal focus, emotional control, positive cognition, family support, and interpersonal assistance. This scale reflects the effectiveness of adolescents’ cognition, emotion, behavior, and environment in helping them resist adversity and adapt well to adversity situations. It is suitable for measuring the psychological resilience of Chinese adolescents. The Cronbach's α coefficient for the total scale is 0.925.

#### Physical Self-efficacy Scale (PSES).

The scale was compiled by Dong [39] et al., using the Likert 5-level scoring method, and was composed of 22 items, including two subscales: body perception ability and physical ability confidence. Higher scores indicate higher levels of physical self-efficacy in middle school students. The Cronbach's α coefficient of the total scale is 0.843.

### Test of common method bias

Since the data collected in this study were all derived from subjective questionnaires, a common method bias test was performed. The results of Harman's one-way test showed that there were 17 factors with eigenvalues greater than 1, and the maximum factor variance explanation rate was 31.06%, which was lower than the 40% critical standard. Therefore, there is no serious common method bias in the data of this study. Harman’s single-factor test was adopted to examine common method bias. The first common factor explained only 31.06% of the total variance, below the 40% critical threshold, which rules out severe common method bias as the cause of this high correlation

### Statistical analysis

This study mainly adopted SPSS 27.0 (authorized and purchased by Nantong University Library) and used Excel software to process the data.The data used in this study were collected at a single time point, forming a cross-sectional design. The predictor variables, mediating variables, and outcome variables were all evaluated simultaneously in November 2024 to ensure temporal consistency. Data processing includes the following steps: (1) Preprocess the data obtained from Questionnaire Star using Excel software, and remeasure or delete the missing or problematic data. (2) Use Harman's single-factor method to evaluate the common method bias. (3) Partial correlation analysis was used to explore the relationship among the principal variables. (4) Regression analysis was used for mediating effect analysis. A specific analysis of the mediating effect of Model 6 was conducted based on the Process plugin, with the confidence interval set at 95%. The Bootstrap method was adopted to analyze the mediating effect in order to explore the correlational interaction mechanism among variables.

## Results

The scores of the variables of the CPEH Curriculum psychological resilience, physical self-efficacy, and psychological stress of junior middle school students were statistically analyzed, and the results are shown in Table 1. There were statistically significant differences in the CPEH Curriculum evaluation scores, psychological resilience scores, and physical self-efficacy scores in terms of gender, and the scores of boys were significantly higher than those of girls. There were statistically significant differences in the CPEH Curriculum evaluation scores, psychological resilience scores, and physical self-efficacy scores in terms of grades (*P* < 0.001). There was no significant difference in the psychological stress score of junior middle school students in terms of gender and grade (*P* > 0.05).

**Table 1:** Descriptive statistics with T-test and ANOVA results.

		CPEH Curriculum		ARS		PSES		MSSSS	
		Mean	SD	Mean	SD	Mean	SD	Mean	SD
Gender	Male	39.00	6.68	98.85	20.7	83.18	13.45	70.5	41.84
	Female	34.08	6.98	103.40	15.42	78.3	12.41	69.58	32.22
	*t*	7.16		−2.47		3.72		0.264	
	*P*	<0.001		0.014		<0.001		0.792	
Grade	7th	34.50	6.90	96.04	14.98	77.0	11.09	71.6	35.27
	8th	37.83	7.40	106.17	18.80	84.4	14.82	65.24	32.25
	9th	37.32	7.00	101.05	19.87	80.4	11.90	74.45	44.70
	*F*	8.78		11.463		12.10		2.11	
	*P*	<0.001		<0.001		<0.001		0.122	

CPEH Curriculum = Evaluation score of the CPEH Curriculum; ARS = Adolescent Resilience Scale; PSES = Physical Self-Efficacy Scale; MSSSS = Middle School Student Stress Scale

Table 2 presents the mean, standard deviation, and correlation coefficients between the CPEH Curriculum, psychological resilience, physical self-efficacy, and psychological stress of junior middle school students. The correlation results showed that psychological stress was significantly negatively correlated with psychological resilience and physical self‑efficacy. The zero-order correlation between the CPEH Curriculum and psychological stress was weakly negative. Significant positive correlations were observed among the CPEH Curriculum, psychological resilience, and physical self-efficacy. Overall, the four variables were significantly correlated, satisfying the statistical prerequisites for cross-sectional-based mediation analysis. We supplemented theoretical explanation for the high correlation r = 0.852 between the CPEH Curriculum and physical self-efficacy. Harman’s single-factor test was adopted to examine common method bias. The first common factor explained only 31.06% of the total variance, below the 40% critical threshold, which rules out severe common method bias as the cause of this high correlation. We further adopted the latent unmeasured variable method to re-examine common method bias. After controlling the common latent factor, the correlation coefficients of all variables did not change significantly, which further ruled out the interference of common method bias on the research results. Although the correlation between CPEH Curriculum and physical self-efficacy was relatively high (r = 0.852), the VIF values of all variables in the regression model were lower than the critical value of 5, indicating no serious multicollinearity. Correlation coefficients only reflect zero-order bivariate relationships, while standardized β values represent independent predictive effects after controlling other variables in the regression model, so numerical inconsistencies between correlation and regression results are reasonable. The two scales measure distinct theoretical constructs, and their essential differences will be elaborated in the Discussion section.

**Table 2:** Correlation analysis of variables.

	Mean	SD	CPEH Curriculum	ARS	PSES	MSSSS
CPEH Curriculum	36.51	7.26	1			
ARS	101.15	18.34	0.30**	1		
PSES	80.72	13.21	0.85**	0.55**		
MSSSS	70.06	37.42	−0.11*	−0.34**	−0.26**	1

*Note:* * *P* < 0.05, ** *P* < 0.01, ***P < 0.001, the same below. CPEH Curriculum = Evaluation score of the CPEH Curriculum; ARS = Adolescent Resilience Scale; PSES = Physical Self-Efficacy Scale; MSSSS = Middle School Student Stress Scale

SPSS 27.0 and the Model 6 model of the PROCESS program developed by Hayes (2013) were used to process and analyze the data. The bias-corrected non-parametric percentile Bootstrap method was used to replicate sampling 5000 times randomly, and the 95% confidence interval of the mediation effect was estimated. The results showed in Table 3 that the CPEH curriculum model showed a significant positive correlational linkage with the psychological resilience of junior middle school students.The CPEH curriculum model and psychological resilience showed significant positive correlational linkages with the physical self-efficacy of junior middle school students.

**Table 3:** Regression analysis of the chain mediation model of psychological resilience and physical self-efficacy.

Dependent variable	Independent variable	*R*	*R* ^2^	*β*	*t*	*P*
ARS		0.300	0.090			<0.001
	CPEH Curriculum			0.300	6.234	<0.001
PSES		0.906	0.815			<0.001
	CPEH Curriculum			0.755	33.766	<0.001
	ARS			0.323	14.443	<0.001
MSSSS		0.372	0.138			<0.001
	CPEH Curriculum			0.229	2.350	0.019
	ARS			−0.234	−3.844	<0.001
	PSES			−0.326	−2.933	0.004

CPEH Curriculum = Evaluation score of the CPEH Curriculum; ARS = Adolescent Resilience Scale; PSES = Physical Self-Efficacy Scale; MSSSS = Middle School Student Stress Scale

In this cross-sectional sample, the CPEH curriculum model showed a significant positive correlational linkage with the psychological stress of junior middle school students.Psychological resilience and physical self-efficacy showed significant negative correlational linkages with the psychological stress of junior middle school students.

The results of the further mediating effect test showed that the 95% confidence interval of the direct effect of the CPEH Curriculum on the psychological stress of students did not include 0, indicating that the direct effect of this pathway was significant. The confidence interval for the indirect effect of the CPEH Curriculum→ psychological resilience → psychological stress in junior middle School Students did not contain 0, indicating that psychological resilience had a significant mediating role in the correlational linkage between the CPEH Curriculum and the psychological stress of students. The confidence interval for the indirect effect of the CPEH Curriculum→ physical self-efficacy → psychological stress in junior middle School Students did not contain 0, indicating that physical self-efficacy had a significant mediating role in the correlational linkage between the CPEH Curriculum and the psychological stress of students. The confidence intervals for the indirect effect of the CPEH Curriculum→ psychological resilience → physical self-efficacy → psychological stress Students also did not contain 0, indicating that psychological resilience and physical self-efficacy had a significant chain mediating role in the correlational linkage between the CPEH Curriculum and the psychological stress of students. In summary, psychological resilience and physical self-efficacy played a mediating role in the correlational linkage between the CPEH Curriculum and psychological stress among students, as shown in Table 4 and Fig 2. All indirect paths had negative effect values and 95% confidence intervals, while the direct path showed positive values, which was consistent with the correlation trend in Table. We calculated the f^2^ effect size of each predictor. The predictive effect of the CPEH Curriculum on physical self-efficacy reached a large effect size, while other paths showed small-to-medium effect sizes.

**Table 4:** The mediating effect of psychological resilience and physical self-efficacy.

	Impact path	Effect size	95% Confidence interval	
			Lower bound	Upper bound
Direct effect	CPEH Curriculum→MSSSS	0.229	0.192	2.155
Indirect effect	CPEH Curriculum→ARS→MSSSS	−0.070	−0.124	−0.026
	CPEH Curriculum→PSES→MSSSS	−0.246	−0.413	−0.073
	Curriculum→ARS→PSES→MSSSS	−0.032	−0.057	−0.009
Total effect		−0.119	−1.118	−0.107

**Fig 2:**
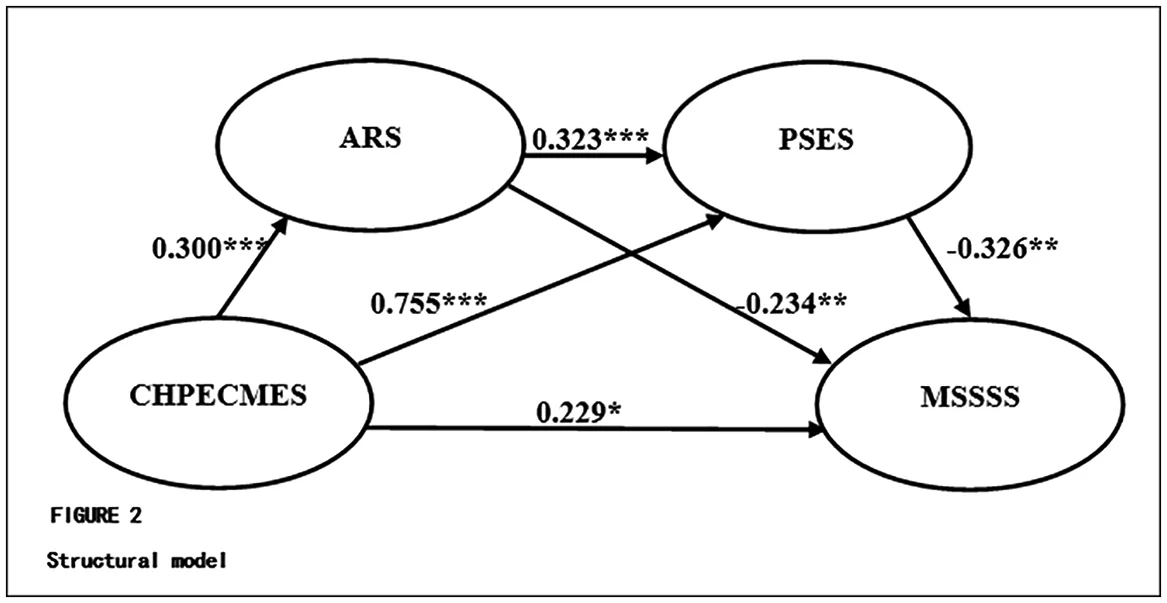
Observed statistical chain-mediating pattern among the CPEH curriculum model, psychological resilience, physical self-efficacy and psychological stress among junior middle school students. All path coefficients displayed in this model are standardized estimates. CPEH Curriculum = Evaluation score of the CPEH Curriculum; ARS = Adolescent Resilience Scale; PSES = Physical Self-Efficacy Scale; MSSSS = Middle School Student Stress Scale.

As shown in Tables 3 and 4, three types of correlational paths are identified, among which psychological resilience’s separate mediation contributes most, and total indirect correlation is weaker than the direct path.

## Discussion

The results of this study showed that there were gender and grade differences in the scores of the evaluation of the CPEH Curriculum, psychological resilience, and physical self-efficacy among students. The scores of boys in the CPEH Curriculum and physical self-efficacy were significantly higher than those of girls, which was consistent with the results of previous studies [29,40]. However, the psychological resilience score of girls is significantly higher than that of boys, which is not completely consistent with the results of existing studies [41]. While some studies have shown that boys have a higher tendency to control than girls [42], another study has shown that boys have higher scores on psychological resilience than girls [43]. Therefore, the conclusion that girls have higher psychological resilience scores than boys remains controversial. The correlation coefficient between CPEH Curriculum perception and physical self-efficacy reaches r = 0.852, which needs reasonable theoretical interpretation. The CPEH scale mainly reflects students’ subjective experience of classroom training, exercise load and curriculum arrangement, while physical self-efficacy represents individual confidence in sports competence. Systematic standardized skill training and in-class competitions under the CPEH Curriculum continuously improve students’ athletic ability and self-recognition, which logically forms a strong positive correlation. Nevertheless, the two constructs are independent without serious overlap: one reflects curriculum implementation experience, and the other belongs to individual sports psychological cognition. Combined with Harman single-factor test results, we confirm that the high correlation is not induced by confused measurement or serious common method bias.

Notably, all associations reported herein are derived from cross-sectional single-wave data; accordingly, we cannot draw definitive causal conclusions that the CPEH Curriculum leads to elevated stress. Existing literature generally confirms that voluntary, self-organized after-school physical activity presents negative synchronous correlations with adolescent psychological stress, which forms the theoretical basis for our initial Hypothesis H1. Nevertheless, such research conclusions cannot be mechanically extended to mandatory school PE courses with unified assessment standards under China’s local educational context. Combined with academic stress superposition theory and evaluation anxiety theory, the CPEH Curriculum belongs to compulsory, assessment-oriented classroom sports, fundamentally different from free leisure exercise. Uniform scoring indicators, mandatory skill examinations and intensive integrated training will overlay additional assessment-related pressure on junior high students on top of daily academic stress. This theoretical system provides systematic grounds to explain why the statistical correlation between CPEH Curriculum and psychological stress is positive, which conflicts with our original research hypothesis.Correlation and regression analysis revealed a significant positive predictive association between the CPEH Curriculum and students’ psychological stress, which fails to support Hypothesis H1. This statistical concurrent linkage cannot be interpreted as a causal impact of the curriculum on stress levels. It should be noted that the following potential influencing factors were not directly measured in the current research, and these explanations are only reasonable speculations summarized from previous relevant studies rather than empirical conclusions supported by our own data.The reasons may be: (1) the pressure of the CPEH Curriculum assessment: The stringent unified evaluation criteria of the CPEH Curriculum show a statistical correlation with students’ worries about failing assessments and subsequent self-reported stress symptoms [44]; (2) Students’ expectation gap: The high standardized learning targets set by the curriculum are associated with a perceived ability-expectation gap, which coincides with higher self-reported psychological stress among respondents [45]; (3) Tight time arrangement: The classroom teaching of the CPEH Curriculum includes technical movement learning, physical exercises, sports competitions and other contents. Compact classroom schedules integrating skill practice, fitness drills and in-class competitions are associated with students’ subjective feelings of psychological burden [18].

Psychological resilience plays a mediating role in the relationship between the CPEH Curriculum and the psychological stress of students, which validates the hypothesis H2 proposed in this study. This result also supports the psychological regulation theory that physical activity covaries with lower the psychological stress of students. From a statistical predictive perspective, higher scores on the CPEH Curriculum evaluation scale correlate with stronger psychological resilience among participants.Previous studies have shown that the CPEH Curriculum emphasizes the subjective position of students, focuses on cultivating students’ sports interests and hobbies, encourages students to actively participate in classroom discussions and interactions, and shifts their attention from stressors such as learning and life to physical activity itself, to higher the psychological resilience [2]. On the other hand, psychological resilience can negatively predict the psychological stress of students. In China's current further education system, test scores are often seen as a key indicator of student’s academic achievement and future development. Anxiety and depression become the main source of stress for students who face frequent and important exams [46], and higher psychological resilience scores are concurrently associated with weaker self-reported stress and negative emotional experiences [47]. In addition, through the implementation of the CPEH Curriculum, students can establish good relationships with their classmates, obtain peer support and emotional communication in physical education classroom activities, and this social interaction can provide emotional support and support for students, which helps to lower psychological stress [48]. All significant mediating paths identified here reflect statistical covariation rather than proven causal chains; longitudinal intervention research is required to verify whether cultivating resilience can actually reduce stress linked to PE participation.

Physical self-efficacy plays a mediating role in the relationship between the CPEH Curriculum and the psychological stress of students, which validates the hypotheses H3 and H4, and supports the self-efficacy covariation theory of physical activity to lower psychological stress. The CPEH Curriculum can significantly predict physical self-efficacy positively, and physical self-efficacy can significantly predict psychological stress in students. It has been found that physical activity 3 times a week for 8–10 min at an average heart rate of 150 bpm for 5 weeks is effective in correlated with higher self-confidence and body self-perception in adolescents [49]. Since the CPEH Curriculum can provide a variety of physical exercises, motor skills learning, and competition activities, it is statistically associated with better body awareness, motor skill mastery and elevated physical self-efficacy [50]. When students feel that they are empowered and confident in physical activity, they are more confident in coping with academic and social pressures [51]. Studies have shown that students with high physical self-efficacy may be more willing to actively participate in physical activity, and regular sports engagement is correlated with endorphin release and improved emotional states, which covary with lower stress scores in this sample [52]. These correlational patterns cannot confirm that shifts in physical self-efficacy directly produce lower self-reported stress; they merely demonstrate simultaneous linkage among the measured variables at one time point.

From a statistical perspective, the CPEH Curriculum exhibits a serial predictive association with students’ psychological stress via psychological resilience and physical self-efficacy, which supports Hypothesis 5 of the present study. The exercise load and competitive activities embedded in the CPEH Curriculum are correlated with students’ experiences of overcoming classroom challenges, greater frustration tolerance, and stronger mental toughness, alongside higher self-perception and confidence regarding their physical capacities. Participants with higher psychological resilience and physical self-efficacy tend to report stronger coping resources and lower concurrent psychological stress symptoms. Accordingly, the present findings align with psychological regulation and self-efficacy theories regarding covariation between physical education experiences and adolescents’ mental status, and illustrate the sequential correlational pattern of psychological resilience and physical self-efficacy, offering tentative references for subsequent research on physical activity and adolescent stress. The overall serial mediation model predicting psychological stress presented a medium effect (f^2^ = 0.16).It is critical to emphasize that the observed serial chain mediation only describes a sequential correlational pattern captured by one-time questionnaire data. We cannot confirm a directional causal pathway in which CPEH Curriculum participation sequentially associates with elevated resilience, then physical self-efficacy, and ultimately diminished stress. Reverse or reciprocal relationships between these psychological variables are also plausible and cannot be ruled out under a cross-sectional design. Future longitudinal tracking or controlled teaching interventions are necessary to disentangle directional causal effects. It should be emphasized that all statistically significant pathways in this study presented relatively small effect sizes. Statistical significance only reflects stable correlations within the sample, while weak effect magnitudes indicate the CPEH Curriculum can only explain limited variance in students’ psychological stress. The cross-sectional design, lack of a conventional PE control group and unmeasured moderators together lead to small effects, so we should not overestimate the correlation intensity between curriculum experience and adolescent stress.

Physical education curricula in the US and Europe differ greatly from the CPEH Curriculum in structure and content. Foreign PE courses focus on optional sports and loose assessment, while the CPEH Curriculum includes mandatory unified evaluations and systematic skill training. Studies from Western scholars generally agree that exercise correlates with higher resilience and physical self-efficacy, but most of them report PE links to lower students’ stress, which contradicts our result that the CPEH Curriculum positively predicts psychological stress. We speculate that this cross-national inconsistency in research results may be linked to divergent assessment mechanisms and educational contextual pressure rather than representing a definitive causal driver.Foreign students face little risk of failing PE, whereas unified grading standards in the CPEH Curriculum bring unique academic stress to domestic students.

This study still has several obvious limitations worthy of attention. First, this study relies entirely on cross-sectional questionnaire data collected at a single time point. While serial mediation models are statistically permissible for such datasets, this design inherently eliminates the ability to verify causal directional effects between variables. We can only interpret correlational and predictive associations rather than draw causal inferences about how the CPEH Curriculum shapes students’ mental status. Longitudinal follow-up investigations or randomized intervention teaching experiments can be designed in future Hstudies to further verify potential causal mechanisms.Longitudinal follow-up investigations or intervention teaching experiments can be designed in future studies to further verify the research conclusions. Second, the research sample only covers domestic junior high Students, and follow-up research can expand the scope to include senior high Students for comparative analysis. Third, this study uses non-probabilistic convenience sampling, which may affect the representativeness of the sample and reduce the generalizability of the research findings to broader student groups. Fourth, this survey only recruited classes adopting the CPEH Curriculum, and no control group with conventional general physical education was arranged. Without a comparison group, we cannot separate the independent correlational characteristics related to the curriculum model, and confounding factors such as campus atmosphere, teachers’ teaching styles and students’ original exercise habits cannot be excluded. The lack of a control group significantly limits the generalizability of our results; the correlational rules observed in this sample cannot be generalized to all junior high school students or schools with different PE arrangements. Future research can set up a control group receiving regular PE courses for comparative analysis. Furthermore, socioeconomic status may affect adolescents’ psychological resilience and stress levels. This study only collected relevant data without further analysis, and future studies could treat socioeconomic factors as control variables.

## Conclusion

Correlation analysis shows that the CPEH Curriculum is significantly correlated with Students’ psychological resilience, physical self-efficacy and psychological stress. In this cross-sectional sample, the CPEH curriculum model showed a significant positive correlational linkage with students’ psychological stress. Psychological resilience and physical self-efficacy play a serial chain mediating role through three pathways: the independent mediating effect of psychological resilience, the independent mediating effect of physical self-efficacy, and the chain mediating effect of the two variables. Notably, all the above pathways and associations are purely statistical predictive relationships derived from cross-sectional data. No causal directional effects can be firmly established in the current study; we cannot conclude that engagement with the CPEH Curriculum itself directly alters students’ psychological stress, resilience or physical self-efficacy.

Although the correlational nature of cross-sectional data prevents us from claiming causal intervention effects, the observed variable associations still provide tentative instructional references for physical education practice centered on the CPEH Curriculum.These findings carry clear practical implications for physical education teaching centered on the CPEH Curriculum. First, schools and PE teachers can appropriately optimize the rigid unified assessment standards of the CPEH Curriculum to potentially ease the assessment-related stress symptoms correlated with rigid unified evaluation standards. Second, more classroom activities focused on cultivating psychological resilience can be arranged to help Students cope with study and sports pressure. Third, diversified sports practice and competitive tasks can be reasonably allocated to continuously sustain higher students’ physical self-efficacy, which may help lower the psychological stress that covaries with CPEH classroom participation. Targeted adjustments to curriculum evaluation and classroom arrangement can balance the training objectives of the CPEH Curriculumand Students’ mental health development. Considering the research limitations elaborated in the Discussion section, the adoption of non-probabilistic sampling also restricts the generalization of the above conclusions to broader student groups.

## Supporting information

S1 Dataset Full anonymized English dataset of participants used in this research. (XLSX)
