# Supplementary material for: Associations between the Chinese physical education and health Curriculum model to the psychological stress of junior middle school students: The chain mediating role of psychological resilience and physical self-efficacy
Source: PLoS One. 2026 Oct 26;21(10):e0360804. doi: 10.1371/journal.pone.0360804 (PMC13704845; doi:10.1371/journal.pone.0360804)
Supplement: Supplementary file 1 [file pone.0360804.s002.docx]

***Title***

Revise the title. I suggest:

*Effects of Chinese physical education and health curriculum model to the psychological stress of junior middle school students: the chain mediating role of psychological resilience and physical self-efficacy*

***Abstract***

Stylistically fix the abstract. Avoid repetition. Remove redundant phrases and omit numerical statistics (r, p) because they have no place there (whoever wants to can see them in the results section).

**Introduction**

OK.

I suggest that the oft-repeated phrase "*Chinese Physical Education and Health*" *Curriculum Model* be replaced with the abbreviation **CPEH** Curriculum and applied throughout the text.

**~~Participants and research methods~~** Correct section title. I suggest just: **Method**

***Study design***

New section. Within this section, describe the research procedure concisely. Mention the permission of the ethics committee and indicate whether the research was conducted in accordance with the Declaration of Helsinki.

***~~Participants~~*** It is better to use the title ***Sample***

- Describe the sampling method. State whether the sample is probabilistic or non-probabilistic (cluster, purposive, etc.).
- Move the data on the start and end of the study to a new section – *Study design*.

***Measuring tools***

*The Chinese Physical Education and Health Curriculum Model Evaluation Scale*

OK.

*The Stressor Scale for Middle School Students*

OK.

*The Resilience Scale for Chinese Adolescents*

OK.

*Physical Self-efficacy Scale*

OK.

***Test of Common Method Bias***

OK.

***~~Statistical Methods~~*** It is better to use the title ***Statistical Analysis***

This study mainly adopted SPSS 27.0 (Specify the Software License) and used Excel software to process the data. The data used in this study were collected at a single time point, forming a cross-sectional design. The predictor variables, mediating variables, and outcome variables were all evaluated simultaneously in November 2024 to ensure temporal consistency. Data processing includes the following steps: (1) Preprocess the data obtained from Questionnaire Star using Excel software, and remeasure or delete the missing or problematic data. (2) Use Harman's single-factor method to evaluate the common method bias. ~~The analysis revealed seventeen major components with eigenvalues greater than 1, among which the largest factor explained 31.06% of the variance. This value is lower than the usually accepted 40% threshold, indicating that the common method bias is not a significant issue in this study.~~ (Delete because this has already been said) (3) Partial correlation analysis was used to explore the relationship among the principal variables. ~~The results showed that the psychological stress of middle school students was significantly negatively correlated with the Chinese health and physical education curriculum model, psychological resilience, and physical self-efficacy. There is a significant positive correlation among the Chinese health and physical education curriculum model, psychological resilience, and physical self-efficacy, that is, there are significant pairwise correlations among these four variables.~~ (Delete. This data belongs to the Results section) (4) Regression analysis was used for mediating effect analysis. A specific analysis of the mediating effect of Model 6 was conducted based on the Process plugin, with the confidence interval set at 95%. The Bootstrap method was adopted to analyse the mediating effect in order to explore the mechanism of interaction among variables.

**Results**

*~~Basic information about each variable~~* (Superfluous inter-title. Delete.)

The scores of the variables of the ~~Chinese physical education and health curriculum model~~ CPEH curriculum psychological resilience, physical self-efficacy, and psychological stress of junior middle school students were ~~descriptively and~~ statistically analysed, and the results are shown in Table 1. There were statistically significant differences in the ~~Chinese physical education and health curriculum model~~ CPEH curriculum evaluation scores, psychological resilience scores, and physical self-efficacy scores in terms of gender ~~(P<0.001)~~, (This is visible in the table and is redundant information in the text) and the scores of boys were significantly higher than those of girls. There were statistically significant differences in the ~~Chinese physical education and health curriculum model~~ CPEH curriculum evaluation scores, psychological resilience scores, and physical self-efficacy scores in terms of grades (P<0.001). ~~The evaluation scores of the eighth and ninth-grade students in the Chinese physical education and health curriculum model were higher than those of the seventh grade. The psychological resilience and physical self-efficacy score of the eighth grade was highest, the ninth grade was second, and the seventh grade was third.~~ (This can be seen in the table and there is no need to duplicate this information in the text.) There was no significant difference in the psychological stress score of junior middle school students in terms of gender and grade (P>0.05).

**Table 1** Descriptive statistics ~~of each variable (n=395)~~ with T-test and ANOVA results

- An explanation of abbreviations **must** be provided below the table.
- Create separate columns for the mean and standard deviation. This is an incorrect way to represent the results - e.g. 39.00±6.68 (many people use it even though it is incorrect). The ± symbol is justified when reporting the Standard Error (and not the Standard Deviation). So, separate the *Mean* column and the SD column.

*~~A chain mediation model test on the influence of the Chinese physical education and health curriculum model on the psychological stress of junior middle school students~~* (Superfluous inter-title. Delete.)

**Table 2** Correlation analysis of variables ~~(n=395)~~ (Delete. This data already exists in the text.)

- An explanation of abbreviations **must** be provided below the table.
- This is how the Mean and SD should be displayed in Table 1. Instead of the abbreviation "M", write *Mean*.

~~Chinese physical education and health curriculum model~~, CPEH curriculum, psychological resilience, physical self-efficacy, and psychological stress of junior middle school students. The results showed a significant negative correlation between the psychological stress of junior middle school students and the mode of physical education and health curriculum, psychological resilience, and physical self-efficacy in China ~~(r = -0.119, -0.345, -0.260, P < 0.01)~~. (This can be seen in the table and there is no need to duplicate this information in the text) There was a significant positive correlation between the mode of physical education and health curriculum, psychological resilience, and physical self-efficacy ~~(r = 0.300, 0.852, 0.550, P < 0.01)~~ (This can be seen in the table and there is no need to duplicate this information in the text), that is, there was a significant correlation between these four variables, which met the requirements of mediating effect analysis. ~~SPSS 23.0 and the Model 6 model of the PROCESS program developed by Hayes (2013) were used to process and analyze the data. The bias-corrected non-parametric percentile Bootstrap method was used to replicate sampling 5000 times randomly, and the 95% confidence interval of the mediation effect was estimated.~~ (This is information that belongs in the Statistical Analysis section and does not need to be repeated in the Results section.) The results showed in Table 3 that the ~~Chinese physical education and health curriculum model~~ CPEH curriculum had a significant positive predictive effect on the psychological resilience of junior middle school students ~~(β =0.300, P < 0.001)~~. (This can be seen in the table and there is no need to duplicate this information in the text.) The curriculum model and psychological resilience had a significant positive predictive effect on the physical self-efficacy of junior middle school students ~~(β = 0.755, 0.323, P < 0.001)~~. (This can be seen in the table and there is no need to duplicate this information in the text.) ~~The Chinese physical education and health curriculum model~~ CPEH curriculum had a significant positive predictive effect on the psychological stress of junior middle school students ~~(β = 0.229, P < 0.05)~~. (This can be seen in the table and there is no need to duplicate this information in the text.) Psychological resilience and physical self-efficacy had a significant negative predictive effect on the psychological stress of junior middle school students ~~(β = -0.234, -0.326, P < 0.01)~~. (This can be seen in the table and there is no need to duplicate this information in the text.)

**Table 3** …. An explanation of abbreviations **must** be provided below the table.

The results of the further mediating effect test showed that the 95% confidence interval of the direct effect of the ~~Chinese physical education and health curriculum model~~ CPEH curriculum on the psychological stress of ~~junior middle school~~ (It is enough to just say "Students" because it is already clear to the reader what age group is involved) students did not include 0 ~~[0.192, 2.155]~~, (This can be seen in the table and there is no need to duplicate this information in the text.) indicating that the direct effect of this pathway was significant ~~(effect size 0.229)~~. (This can be seen in the table and there is no need to duplicate this information in the text.) The ~~95%~~ (It's implied because it's already been said) confidence interval for the indirect effect of the ~~Chinese physical education and health curriculum model~~ CPEH curriculum → psychological resilience → psychological stress in junior middle School Students did not contain 0 ~~[-0.124, - 0.026]~~, (This can be seen in the table and there is no need to duplicate this information) indicating that psychological resilience had a significant mediating role in the effect of the curriculum model on the psychological stress of junior middle school students ~~(the value of the mediating effect was -0.070, accounting for 20.11% of the total indirect effect)~~. (This can be seen in the table and there is no need to duplicate this information) The ~~95%~~ (It's implied because it's already been said) confidence interval for the indirect effect of the ~~curriculum model~~ CPEH curriculum → physical self-efficacy → psychological stress in ~~junior middle School~~ (It is enough to just say "Students" because it is already clear to the reader what age group is involved) Students did not contain 0 ~~[-0.413, -0.073]~~, (This can be seen in the table and there is no need to duplicate this information) indicating that physical self-efficacy had a significant mediating role in the effect of the curriculum model on the psychological stress of junior middle school students ~~(the value of the mediating effect was -0.246, which accounted for 70.69% of the total indirect effect)~~. (This can be seen in the table and there is no need to duplicate this information) The ~~95%~~ confidence intervals for the indirect effect of the Chinese physical education and health ~~curriculum model~~ CPEH curriculum → psychological resilience → physical self-efficacy → psychological stress in junior middle School Students also did not contain 0 [-0.057, -0.009], indicating that psychological resilience and physical self-efficacy had a significant chain mediating role in the effect of the curriculum model on the psychological stress of ~~junior middle school~~ students ~~(the value of the mediating effect was -0.032, which accounted for 9.20% of the total indirect effect)~~. In summary, psychological resilience and physical self-efficacy played a mediating role in the effect of the Chinese physical education and health curriculum model on psychological stress among junior middle school students ~~(total effect value of -0.119)~~, as shown in Table 4 and Figure 2.

**Table 3** …. An explanation of abbreviations **must** be provided below the table.

**Important general note:**

One of the rules of writing scientific papers is that data should not be repeated frequently. What is reported in Tables should not be cited in the text; what is described in the text should not be repeated in Figures, etc.

**Discussion**

- The discussion is written logically and clearly. However, a comparison of the Chinese curriculum model with some other national models (e.g. USA or EU) is missing. Such a comparative analysis would increase the quality of the paper.
- I suggest writing 1-2 paragraphs about it and answering the following questions: (1) Are the structure and content of curricula in other countries the same or different from the CPES? (2) Are the findings of US and EU researchers consistent with the findings of this study? (3) If the findings are not the same, how can the differences be explained?
- Use the previously proposed abbreviation CPES curriculum throughout the discussion.
- There is no need to always repeat the information about the age of the participants; it is enough to say only Students (the reader will surely already understand which category of students is being discussed).

**Conclusion**

- The conclusion only repeats the most important results. The goal of the conclusion is not just to repeat what has already been said, but primarily to recommend the authors how their findings can be applied in didactic practice.
- The review of the limitations of the study (last 3-4 lines) should be moved to the end of the Discussion. In addition to the aforementioned limitations, it is important to also mention the impact of non-probabilistic sampling on the generalizability of the results obtained.

**References**

OK.
