# Supplementary material for: Associations between the Chinese physical education and health Curriculum model to the psychological stress of junior middle school students: The chain mediating role of psychological resilience and physical self-efficacy
Source: PLoS One. 2026 Oct 26;21(10):e0360804. doi: 10.1371/journal.pone.0360804 (PMC13704845; doi:10.1371/journal.pone.0360804)
Supplement: Supplementary file 2 [file pone.0360804.s003.docx]

Manuscript Title: The relationship between the Chinese physical education and health curriculum model and the psychological stress of junior middle school students: the chain mediating role of psychological resilience and physical self-efficacy.

General Assessment:

The manuscript addresses an important topic—the role of structured physical education in mitigating psychological stress among Chinese junior middle school students. The research design includes appropriate psychological measures and statistical analyses to test the hypothesized chain mediation model. The study provides potentially valuable insights into how curriculum-based physical education influences student well-being via psychological resilience and physical self-efficacy.

However, there are notable concerns regarding study design, statistical interpretation, clarity of hypotheses, and presentation of results that need to be addressed before publication.

Major Comments:

1. Study Design and Temporal Interpretation:

-The study is cross-sectional, which limits causal inference. While the chain mediation model is appropriate statistically, the language used in the discussion and conclusion often implies causality (e.g., “the curriculum model can alleviate psychological stress”), which is not strictly supported. Consider rephrasing to reflect correlational rather than causal conclusions.

- The survey period mentions a start date of February 31, 2025, which is a non-existent date and should be corrected.

2. Hypothesis Consistency and Interpretation:

- Hypothesis H1 predicted that the curriculum model would negatively predict psychological stress; however, the results indicate a positive relationship. The discussion acknowledges this but could be more rigorous in exploring possible confounding factors and alternative explanations.

- The manuscript sometimes conflates direct and indirect effects without clear distinction. Tables 3 and 4 report both direct and indirect effects, but the narrative should explicitly describe the relative contributions of each pathway in lay terms for clarity.

3. Statistical Analysis:

- Harman’s single-factor test was used to check for common method bias; while adequate, the reliance solely on this method may be insufficient. Consider complementary tests or controlling for method variance in structural analyses.

- The regression coefficients and β values sometimes appear inconsistent with the correlation results; clarification is needed. Additionally, confidence intervals for some indirect effects are reported as negative ranges with positive effect sizes—this needs correction for clarity and accuracy.

4. Writing, Clarity, and Organization:

- The manuscript is generally understandable but contains long, complex sentences that occasionally obscure meaning. Editing for conciseness and readability is recommended.

- Some figures (Figure 1, Figure 2) and tables lack sufficient description in the legend, making them difficult to interpret without referring extensively to the text.

5. Demographic Details:

- The demographic description could also report socioeconomic status, which may influence psychological stress and resilience.

Minor Comments:

- Correct minor typographical errors, e.g., “February 31, 2025” and inconsistent formatting of citations.

- Standardize terms, e.g., “Chinese physical education and health curriculum model” is sometimes shortened inconsistently.

- Consider providing effect size measures (Cohen’s d, f²) for key relationships to enhance interpretability.

- Improve figure quality and ensure all axes and labels are clear.

Recommendation:

Major Revision: The manuscript presents valuable findings but requires significant revisions in clarity, statistical interpretation, and discussion of causal claims. Emphasis should be placed on:

1. Reframing causal language to correlational interpretations.

2. Clarifying statistical analyses and mediation pathways.

3. Strengthening discussion of unexpected findings (e.g., H1).

4. Improving clarity, grammar, and figue/table presentation.
