# Supplementary material for: Associations between the Chinese physical education and health Curriculum model to the psychological stress of junior middle school students: The chain mediating role of psychological resilience and physical self-efficacy
Source: PLoS One. 2026 Oct 26;21(10):e0360804. doi: 10.1371/journal.pone.0360804 (PMC13704845; doi:10.1371/journal.pone.0360804)
Supplement: Supplementary file 3 [file pone.0360804.s004.docx]

**REVIEWER’S REPORT**

**Article Title:** The relationship between the Chinese physical education and health curriculum model and the psychological stress of junior middle school students: the chain mediating role of psychological resilience and physical self-efficacy

**General Assessment**

This study examines the effect of the Chinese physical education and health curriculum model on psychological stress among secondary school pupils, and the mediating roles of psychological resilience and physical self-efficacy in this relationship. The topic is particularly topical and significant in the context of education policies and adolescent mental health. The sample size is adequate, and the testing of the chain mediation model is one of the study’s strengths.

However, the study contains significant methodological and conceptual issues. As it stands, the interpretation of the findings and the reliability of the conclusions are severely limited. Therefore, major revisions are recommended for the study; in its current form, it is not suitable for publication.

**1. Research Design and the Causality Issue**

The study employs a cross-sectional design, and all variables were measured at a single point in time. Despite this, the article frequently uses causal expressions such as ‘effects’, ‘predicts’, and ‘increases/decreases’.

- Mediation analysis assumes a temporal sequence between variables; this study does not meet this assumption.
- Therefore, the results obtained can be interpreted as correlational.

**Recommendation:** The causal language used throughout the article should be reviewed, and the results should be restated within a correlational framework. This limitation should be clearly stated in the discussion section.

**2. Lack of a Control Group**

The study employed a convenience sampling method and examined nine classes exposed to the same curriculum model.

- The study does not include a control or comparison group.
- This makes it difficult to distinguish the effect of the curriculum model.

**Recommendation:** The authors should clearly state this limitation and emphasise that the generalisability of the results is restricted.

**3. Excessively High Correlation Value**

The study reported a very high level of correlation between certain variables (r = 0.852).

- A correlation of this magnitude suggests that the variables do not sufficiently distinguish from one another.
- There may be a possibility that the scales measure similar constructs or that common method bias is present.

**Recommendation:** The authors should discuss this issue and present additional analyses to support the discriminant validity of the measurements.

**4. Data and Reporting Inconsistencies**

There are inconsistencies in the article regarding the date of data collection (e.g. invalid date formats).

**Recommendation:** All methodological information should be carefully reviewed and corrected.

**Issues Regarding the Discussion Section**

**1. Inconsistency Between Findings and Hypotheses**

One of the study’s main hypotheses is that the curriculum model will reduce stress; however, the results suggest the opposite. The discussion section fails to explain this situation with a sufficiently robust theoretical framework.

**2. Speculative Interpretations**

Some explanations (e.g. course workload, assessment pressure) are based on variables that have not been directly measured. Such interpretations are not evidence-based.

**3. Persistence of Causal Language**

Despite the cross-sectional design, causal language continues to be used in the discussion section.

**4. Insufficient Discussion of Effect Sizes**

Although some relationships are statistically significant, the effect sizes are small. This issue has not been adequately addressed in the discussion.

**Conclusion and Recommendation**

Whilst the article focuses on an important topic, in its current form it contains serious methodological and interpretative issues. In particular, given the limitations of the research design, it is inappropriate to publish the study without addressing the claims of causality. The authors must undertake comprehensive methodological and conceptual revisions.
