# Supplementary material for: Associations between the Chinese physical education and health Curriculum model to the psychological stress of junior middle school students: The chain mediating role of psychological resilience and physical self-efficacy
Source: PLoS One. 2026 Oct 26;21(10):e0360804. doi: 10.1371/journal.pone.0360804 (PMC13704845; doi:10.1371/journal.pone.0360804)
Supplement: Supplementary file 4 [file pone.0360804.s006.docx]

Point-by point response to the comments of Reviewer 5

Q1:

The time report in the study contains significant inconsistencies. The Methods section states that the research began on "February 31, 2025," a date that does not exist. However, the Statistical Methods section claims that all variables were assessed in "November 2024," creating a temporal contradiction. The authors are requested to verify and unify the data collection timeline, as failure to do so will undermine the study's credibility.

Q2:

Insufficient sample information. The authors should specify the number of schools, regions, and classes involved, the sampling method, inclusion and exclusion criteria, and whether all students received the same course model. The current statement of "nine classes, convenience sampling" is insufficient to assess the representativeness of the sample.

Q3:

Abbreviations of variables in the chart should be annotated upon their first occurrence.

Q4:

There are contradictions in the argumentation. Since the higher the score on the middle school students' psychological stress scale indicates a higher level of psychological stress among them, both the results and discussion sections conclude that the Chinese physical education and health curriculum model positively predicts junior high school students' psychological stress and provide explanations. However, the discussion also mentions that "implementing the Chinese physical education and health curriculum model helps alleviate their psychological stress." The conclusion further states that "the curriculum model can significantly and positively predict junior high school students' psychological stress." This creates a contradiction between the results and the discussion.

**Reply:**

# Response to Reviewer #5

We sincerely appreciate the reviewer’s careful and rigorous review and valuable constructive comments. We have revised and supplemented the manuscript item by item in accordance with your suggestions. All revised and newly added text is highlighted in yellow with corresponding line numbers for easy inspection. The detailed revisions are presented as follows:

## Comment 1: Inconsistent and invalid data collection time

The manuscript contains obvious chronological inconsistencies in data collection time. An invalid date “February 31, 2025” was written in the Methods section, while the statistical analysis part stated that all variables were measured in November 2024, which reduces the credibility of this research. The authors need to verify and unify the data collection timeline.

### Response:

We apologize for the careless writing error and conflicting time descriptions in the original manuscript, which seriously undermine the methodological rigor of this study. We have thoroughly proofread and unified all descriptions of data collection time in the Methods section (Lines 123, yellow highlighted).

1.The non-existent invalid date “February 31, 2025” has been completely deleted;

2.The unified official data collection window is revised to November 2024, and all time-related sentences across the full manuscript are standardized to this unified timeline to eliminate chronological contradictions;

3.We double-checked all time descriptions in sampling, data screening and statistical analysis sections to ensure no conflicting time information remains in the revised version.

## Comment 2: Insufficient sample information

Only vague descriptions of nine classes via convenience sampling are provided. The manuscript fails to clarify the number and regions of surveyed schools, detailed class information, specific sampling procedures, inclusion and exclusion criteria, and whether all participants received identical curriculum arrangements. The current brief description cannot support the evaluation of sample representativeness.

### Response：

We fully accept this valuable suggestion. We have supplemented comprehensive and detailed sample information in the Sampling subsection of the Methods section (Lines 125–130, newly added, yellow highlighted). Meanwhile, we integrated the sampling bias limitation with the missing control group limitation in the Limitations section of Discussion (Lines 414–422, yellow highlighted), consistent with our previous revisions:

1.Supplementary regional and school information: We clearly stated the survey city and district, as well as the total number of participating public junior high schools; we specified that all 9 recruited intact Grade 7–9 classes were involved in the survey, and fully described the complete convenience sampling procedure and the selection logic of target schools and classes.

2.Explicit inclusion and exclusion criteria for student participants are supplemented: Inclusion criteria include regular full attendance of CPEH courses and complete questionnaire responses; exclusion criteria include long-term absence from PE classes, largely incomplete questionnaires, and students with diagnosed mental health disorders.

3.Uniform curriculum statement: We explicitly confirm that all nine recruited classes adopted the identical national CPEH Curriculum model with consistent teaching arrangements, class content and assessment standards.

4.Combined limitation explanation together with the absence of a control group: This study only recruited classes implementing the CPEH Curriculum without setting a comparison group receiving conventional general PE. Coupled with inherent selection bias of convenience sampling, we cannot separate exclusive correlational characteristics uniquely linked to the curriculum model, nor rule out confounding interference from campus atmosphere, teachers’ teaching styles, students’ inherent exercise habits and other irrelevant factors. The generalizability of our findings is greatly restricted, and the correlational patterns observed in this sample cannot be arbitrarily generalized to all junior high students or schools with different PE arrangements. We also propose that follow-up research can adopt stratified sampling and set up a regular PE control group to conduct inter-group comparative analysis. All newly supplemented content is located at Lines 125–130 and Lines 414–422, marked with yellow highlight for easy inspection.

## Comment 3: Undefined variable abbreviations in figures and tables

Abbreviations for variables in charts lack full-name annotations at their first appearance.

### Response：

We have supplemented full name explanations for all variable abbreviations throughout the main text and figure captions:

1.Every variable abbreviation (e.g., CPEH, psychological resilience, physical self-efficacy) is matched with its complete full name upon its first mention in the manuscript (Lines 8–11, yellow highlighted);

2.Unified abbreviation notes are added at the bottom of all figure captions and table footnotes, including the serial chain mediation path diagram (Figure 1) and all correlation and regression tables (Table 1–4). All revised captions are yellow highlighted;

3.A full-text sweep was conducted to ensure no undefined abbreviations appear in any paragraphs, tables or figures.

## Comment 4: Contradictory interpretative logic in the manuscript

Higher scores on the adolescent psychological stress scale indicate greater stress. Both the Results and Discussion sections report a significant positive predictive association between the CPEH Curriculum and students’ psychological stress and provide corresponding interpretations. However, the Discussion also contains the statement that “implementing the CPEH Curriculum helps alleviate their psychological stress”, and the Conclusion further claims that “the curriculum model can significantly and positively predict junior high students’ psychological stress”, creating internal logical conflict.

### Response：

We sincerely thank the reviewer for identifying this self-contradictory interpretative logic, which originates from residual inappropriate causal wording inconsistent with the cross-sectional research design. All conflicting sentences have been fully revised and marked yellow highlighted:

1.The contradictory causal sentence in the original Discussion “implementing the CPEH Curriculum helps alleviate their psychological stress” (Lines 348–350, yellow highlighted) is completely rewritten. All causal verbs such as “alleviate” are removed, and replaced with neutral correlational description: cross-sectional data reveals a synchronous positive correlation between participation in the CPEH Curriculum and students’ self-reported psychological stress;

2.All descriptions related to Hypothesis H1 in Results, Discussion and Conclusion are unified: we consistently clarify that the statistically significant positive predictive association merely reflects contemporaneous synchronous linkage, and explicitly emphasize this correlation cannot be interpreted as a causal effect that the curriculum increases or reduces stress;

3.All practical intervention implications in the Conclusion are revised to remove one-way causal claims (Lines 440–445, yellow highlighted). We eliminate all expressions implying the curriculum can relieve stress; all instructional suggestions are framed as tentative references derived from observed correlational patterns, with clear disclaimers that longitudinal and intervention studies are required to verify potential causal effects;

4.We retain the independent theoretical analysis paragraph (Lines 302–315, yellow highlighted) to systematically explain why the empirical positive correlation conflicts with the original Hypothesis H1, eliminating all internal logical contradictions in the manuscript.

## Overall Revision Summary

All four issues raised by Reviewer #5 have been fully addressed. We corrected the invalid date and unified the data collection timeline, supplemented complete sample background and sampling details, added full annotations for all chart abbreviations, and eliminated contradictory interpretative logic induced by improper causal language. All revisions and supplementary content are highlighted in yellow with clear line numbers for your re-evaluation
