# Supplementary material for: Associations between the Chinese physical education and health Curriculum model to the psychological stress of junior middle school students: The chain mediating role of psychological resilience and physical self-efficacy
Source: PLoS One. 2026 Oct 26;21(10):e0360804. doi: 10.1371/journal.pone.0360804 (PMC13704845; doi:10.1371/journal.pone.0360804)
Supplement: Supplementary file 5 [file pone.0360804.s007.docx]

Response to Editorial Comments

Manuscript Title: The relationship between the Chinese physical education and health curriculum model and the psychological stress of junior middle school students: the chain mediating role of psychological resilience and physical self-efficacy

Corresponding Author: Aimin Ma

We have fully revised the manuscript point by point strictly in accordance with all the reviewer comments raised in the first round of revision.

We double-checked the entire manuscript again carefully this time, including research description wording, comparative analysis of curriculum models, and repeated data in tables and figures.

All the suggested modifications have been completely implemented in the revised manuscript and marked clearly for easy review.

We noticed that the system only provides the same round-one reviewer comments without new feedback for this second revision, so we have re-examined all revisions thoroughly once more to guarantee all requirements are fully satisfied. We sincerely ask the editor and reviewers to re-evaluate our work.

We sincerely appreciate the careful editorial review and detailed guidance provided by Guest Editor Jiajia Ye. We have fully revised the manuscript point by point in strict accordance with all nine editorial requirements. The detailed revisions are listed below:

Comment 1. Format the manuscript following PLOS ONE style guidelines and standard file naming rules

Response: We have adjusted the layout, paragraph format, table and figure formatting of the full manuscript to comply with PLOS ONE style requirements, referring to the two official formatting templates provided by the journal. All uploaded files are named following the standard naming rules of Editorial Manager for this revision.

Comment 2. Supplement complete informed consent details in Methods, clarify written/parental consent for minor participants; ethics statement only in Methods

Response: We supplemented complete informed consent descriptions in the Study Design subsection of Methods:

All participants and their parents/legal guardians were fully informed of the study background, procedures and research objectives before data collection;

Formal written informed consent forms were signed by both adolescent students and their guardians;

All ethical descriptions are integrated only within the Methods section, and any redundant ethical paragraphs outside Methods have been fully deleted to meet the journal’s regulations.

Comment 3. Remove all funding content from manuscript body; add complete funding statement to Cover Letter

Response: We have completely deleted all funding-related text in the manuscript’s Acknowledgments and other sections. The formal revised funding statement is added at the end of the Cover Letter as requested:

This work was supported by the General Program of Jiangsu Provincial Social Science Fund (No. 22TYB008): Research on High-quality Development of Primary and Secondary School Physical Education under the Background of the “Double Reduction” Policy, and the 2025 Undergraduate Innovation Training Program of Nantong University: Innovative Practice of Adolescent Precise Weight Management Driven by Digital Sports.

We authorize the editorial office to update the funding information in our online submission form based on the statement in our Cover Letter.

Comment 4. Add funder role statement in Cover Letter for financial disclosure

Response: The standard funder role disclaimer has been appended after the funding description in the Cover Letter:

The funders had no role in study design, data collection and analysis, decision to publish, or preparation of the manuscript.

Comment 5. Complete ORCID ID binding and verification for corresponding author

Response: The corresponding author has logged into Editorial Manager, entered the “Update My Information” page, and completed the acquisition and verification of the ORCID iD via the official ORCID link. The valid ORCID ID has been bound to the submission account.

Comment 6. All ethical statements are restricted to the Methods section only

Response: We double-checked the full text. All ethical approval and informed consent content is placed solely in the Study Design subsection under Methods. Any separate standalone Ethics section at the end of the original manuscript has been removed entirely.

Comment 7. Upload fully translated English anonymized dataset as Supporting Information

Response: All Chinese text in the original dataset (variable labels, scale items, response options, demographic categories) has been fully translated into English. The anonymized English dataset file S1 Dataset is uploaded as Supporting Information in this revision, complying with PLOS ONE’s data availability policy.

Comment 8. Add Supporting Information description at manuscript end and add in-text citations

Response: A dedicated Supporting Information section is added before the reference list at the end of the manuscript:

Supporting Information

S1 Dataset. Full anonymized English participant dataset used for all statistical analyses in this cross-sectional study.

Meanwhile, we added a sentence in the Methods section to cite the dataset: All valid raw data used for analysis are available in S1 Dataset.

Comment 9. Evaluate recommended references from reviewers

Response: We have reviewed all literature recommended by the reviewers. After evaluating their relevance to our research theme, we have incorporated highly relevant papers into the reference list and cited them appropriately in the main text where necessary.

All modifications above are clearly marked in the revised manuscript with track changes for easy inspection. We hope the revised manuscript meets all the publication requirements of PLOS ONE. Thank you very much for your time and guidance.

Sincerely,

Aimin Ma

On behalf of all co-authors
