# Supplementary material for: Associations between the Chinese physical education and health Curriculum model to the psychological stress of junior middle school students: The chain mediating role of psychological resilience and physical self-efficacy
Source: PLoS One. 2026 Oct 26;21(10):e0360804. doi: 10.1371/journal.pone.0360804 (PMC13704845; doi:10.1371/journal.pone.0360804)
Supplement: Supplementary file 6 [file pone.0360804.s008.docx]

Response to Reviewers 3

1. Clarification and consistency of terminology and abbreviations.

Q1:

The manuscript uses several abbreviations and measurement-related terms, including CPEH, PSES, and psychological resilience-related measures. Although these abbreviations are appropriately defined when first introduced, the authors are encouraged to conduct a final review to ensure consistent usage throughout the entire manuscript, including the abstract, main text, tables, and figures.

Response to Q1：

We greatly appreciate this careful comment. We have systematically checked and unified all abbreviations throughout the full manuscript, including the abstract, main text, tables, and figure captions. All scales are clearly defined with their full names and corresponding abbreviations at the first appearance in the Methods section, including CPEH Curriculum, MSSSS, ARS, and PSES. In addition, we corrected spelling errors in Table 2 and removed inconsistent abbreviations to ensure strictly uniform terminology presentation across the entire paper.(Yellow-highlighted revisions: lines 172, 186, 194, 201, and 241)

Q2:

In addition, some overlapping terms, such as “psychological stress,” “stress level,” and “mental stress,” appear to be used interchangeably. These terms may imply different conceptual meanings in psychological research. Therefore, the authors should clarify whether these terms refer to the same underlying construct or represent distinct dimensions. If they refer to the same construct, a unified term should be adopted throughout the manuscript. If differences exist, the conceptual distinctions should be explicitly explained.

Improving terminology consistency would enhance conceptual precision and reduce potential ambiguity for readers.

Response to Q2：

Thank you for this valuable suggestion. All stress‑related outcome variables in this study belong to the same latent construct measured by the Middle School Student Stress Scale (MSSSS). We have fully unified the terminology in the revised manuscript. Specifically, the inconsistent expression “stress levels” has been completely replaced with the standardized term “psychological stress” to avoid conceptual confusion. We have also added an explanatory sentence in the Methods section to clarify that all stress indicators analyzed in this study correspond to the same MSSSS-measured construct. Only descriptive expressions such as “stress symptoms” are retained for situational description without affecting the unity of core variable definitions. Full-manuscript proofreading has been completed to ensure rigorous and consistent terminology usage.(Yellow-highlighted revisions: lines 477,191)

2. Further description of the CPEH Curriculum implementation

The CPEH Curriculum remains a central component of the current study; however, the description of its implementation is still relatively limited. Although the revised manuscript provides additional information, more contextual details would improve the transparency and interpretability of the findings.

The authors are encouraged to further describe:

• the specific components and educational objectives of the CPEH Curriculum;

• the duration, frequency, and overall implementation process;

• teacher qualifications, training procedures, or requirements for curriculum delivery;

• the major differences between the CPEH Curriculum and conventional physical education classes.

Providing additional details would help readers from different educational contexts better understand the intervention characteristics and evaluate the potential applicability and reproducibility of the findings.

Response ：

Thank you for this constructive comment. We agree that sufficient implementation-related descriptions of the CPEH Curriculum are essential to enhance the transparency and reproducibility of this study. Accordingly, we have added a new subsection titled CPEH Curriculum Implementation in the Methods section immediately after Sample and before Measuring tools. Within this subsection, we have elaborated on: (1) the specific core components and educational objectives of the CPEH Curriculum; (2) lesson duration, weekly frequency and the complete standardized implementation procedure for each class; (3) teachers’ professional qualifications, pre‑implementation training, and curriculum delivery supervision requirements; and (4) the key distinctions between the CPEH Curriculum and conventional physical education classes. These supplementary contents allow readers from diverse educational backgrounds to fully grasp the intervention features and assess the applicability and reproducibility of our results.(Modifications are highlighted in yellow at lines 152–170 of the revised manuscript.)

3. Improvement of tables and figures presentation

The tables and figures are generally informative and contribute effectively to the presentation of the results. However, several minor improvements are recommended to enhance clarity and ensure accurate interpretation.

The authors should ensure that:

• all abbreviations used in tables are clearly defined in table notes;

• decimal places are standardized throughout all statistical results;

• figure legends provide sufficient explanation so that figures can be understood independently from the main text.

For the mediation model figure, the authors should explicitly indicate whether the reported path coefficients represent standardized or unstandardized estimates. This information is important for readers to correctly interpret the magnitude and meaning of the reported effects.

Response ：

Thank you very much for your valuable and detailed suggestions to improve the presentation of our tables and figures. We have carefully revised all tables and figures according to your comments to enhance clarity, uniformity, and readability.

First, we have supplemented full definitions for all abbreviations used in each table within their corresponding table notes to clarify variable terminology.

Second, we standardized decimal‑place formatting for all statistical outputs across the manuscript. Means, standard deviations, correlation coefficients, regression coefficients, t-values and F-values are uniformly reported to two decimal places.

Third, we expanded the legends for Figure 1 and Figure 2 by adding model descriptions. The figures can now be fully interpreted independently without referring to the main text.

Fourth, we explicitly state in both figure legends that all path coefficients in the chain-mediation models are standardized estimates, so readers can correctly interpret the magnitude and statistical meaning of each path.(Modifications are highlighted in yellow at lines 239,243,123,315,119,307 of the revised manuscript.)
