# Supplementary material for: Associations between the Chinese physical education and health Curriculum model to the psychological stress of junior middle school students: The chain mediating role of psychological resilience and physical self-efficacy
Source: PLoS One. 2026 Oct 26;21(10):e0360804. doi: 10.1371/journal.pone.0360804 (PMC13704845; doi:10.1371/journal.pone.0360804)
Supplement: Peer review history

# Peer review: Decision Letter 0

**DOI:** 10.1371/journal.pone.0360804.r001

**Authors:** Jiajia Ye

26 May 2026

Dear Dr. Ma,

Thank you for submitting your manuscript to PLOS ONE. After careful consideration, we feel that it has merit but does not fully meet PLOS ONE’s publication criteria as it currently stands. Therefore, we invite you to submit a revised version of the manuscript that addresses the points raised during the review process.

Please submit your revised manuscript by Jul 10 2026 11:59PM. If you will need more time than this to complete your revisions, please reply to this message or contact the journal office at plosone@plos.org. When you're ready to submit your revision, log on to https://www.editorialmanager.com/pone/ and select the 'Submissions Needing Revision' folder to locate your manuscript file.

- A letter that responds to each point raised by the academic editor and reviewer(s). You should upload this letter as a separate file labeled 'Response to Reviewers'.
- A marked-up copy of your manuscript that highlights changes made to the original version. You should upload this as a separate file labeled 'Revised Manuscript with Track Changes'.
- An unmarked version of your revised paper without tracked changes. You should upload this as a separate file labeled 'Manuscript'.

If you would like to make changes to your financial disclosure, please include your updated statement in your cover letter. Guidelines for resubmitting your figure files are available below the reviewer comments at the end of this letter.

If applicable, we recommend that you deposit your laboratory protocols in protocols.io to enhance the reproducibility of your results. Protocols.io assigns your protocol its own identifier (DOI) so that it can be cited independently in the future. For instructions see: https://journals.plos.org/plosone/s/submission-guidelines#loc-laboratory-protocols. Additionally, PLOS ONE offers an option for publishing peer-reviewed Lab Protocol articles, which describe protocols hosted on protocols.io. Read more information on sharing protocols at https://plos.org/protocols?utm_medium=editorial-email&utm_source=authorletters&utm_campaign=protocols.

As the corresponding author, your ORCID iD is verified in the submission system and will appear in the published article. PLOS supports the use of ORCID, and we encourage all coauthors to register for an ORCID iD and use it as well. Please encourage your coauthors to verify their ORCID iD within the submission system before final acceptance, as unverified ORCID iDs will not appear in the published article. *Only*  the individual author can complete the verification step; PLOS staff *cannot*  verify ORCID iDs on behalf of authors.

We look forward to receiving your revised manuscript.

Kind regards,

Jiajia Ye

Guest Editor

PLOS One

Journal Requirements:

When submitting your revision, we need you to address these additional requirements.

1. Please ensure that your manuscript meets PLOS ONE's style requirements, including those for file naming. The PLOS ONE style templates can be found at

https://journals.plos.org/plosone/s/file?id=wjVg/PLOSOne_formatting_sample_main_body.pdf and

https://journals.plos.org/plosone/s/file?id=ba62/PLOSOne_formatting_sample_title_authors_affiliations.pdf

2. Please provide additional details regarding participant consent. In the ethics statement in the Methods and online submission information, please ensure that you have specified (1) whether consent was informed and (2) what type you obtained (for instance, written or verbal, and if verbal, how it was documented and witnessed). If your study included minors, state whether you obtained consent from parents or guardians. If the need for consent was waived by the ethics committee, please include this information.

If you are reporting a retrospective study of medical records or archived samples, please ensure that you have discussed whether all data were fully anonymized before you accessed them and/or whether the IRB or ethics committee waived the requirement for informed consent. If patients provided informed written consent to have data from their medical records used in research, please include this information.

3. Thank you for stating the following in the Acknowledgments Section of your manuscript:

1. General program of Jiangsu Province Social Science Found of China: Research on high-quality development of primary and secondary school sports under the background of “Double Reduction”.  (22TYB008)  2. Nantong University's 2025 College Student Innovation Training Program Project: Innovative Practice of Precision Weight Management for Adolescents Driven by Digital Sports”

We note that you have provided funding information that is not currently declared in your Funding Statement. However, funding information should not appear in the Acknowledgments section or other areas of your manuscript. We will only publish funding information present in the Funding Statement section of the online submission form.

Please remove any funding-related text from the manuscript and let us know how you would like to update your Funding Statement. Currently, your Funding Statement reads as follows:

“General program of Jiangsu Province Social Science Found of China: Research on high-quality development of primary and secondary school sports under the background of “Double Reduction”. (22TYB008) 2. Nantong University's 2025 College Student Innovation Training Program Project: Innovative Practice of Precision Weight Management for Adolescents Driven by Digital Sports”

Please include your amended statements within your cover letter; we will change the online submission form on your behalf.

4. Thank you for stating the following financial disclosure:

“General program of Jiangsu Province Social Science Found of China: Research on high-quality development of primary and secondary school sports under the background of “Double Reduction”. (22TYB008) 2. Nantong University's 2025 College Student Innovation Training Program Project: Innovative Practice of Precision Weight Management for Adolescents Driven by Digital Sports”

Please state what role the funders took in the study.  If the funders had no role, please state: "The funders had no role in study design, data collection and analysis, decision to publish, or preparation of the manuscript."

If this statement is not correct you must amend it as needed.

Please include this amended Role of Funder statement in your cover letter; we will change the online submission form on your behalf.

5. PLOS requires an ORCID iD for the corresponding author in Editorial Manager on papers submitted after December 6th, 2016. Please ensure that you have an ORCID iD and that it is validated in Editorial Manager. To do this, go to ‘Update my Information’ (in the upper left-hand corner of the main menu), and click on the Fetch/Validate link next to the ORCID field. This will take you to the ORCID site and allow you to create a new iD or authenticate a pre-existing iD in Editorial Manager.

6. Your ethics statement should only appear in the Methods section of your manuscript. If your ethics statement is written in any section besides the Methods, please delete it from any other section.

7. Thank you for providing your underlying data as Supporting Information.

We note that the data set contains text or data that is not in English. Please note that PLOS is an English-language publisher, so we require data sets to be provided in English as well. Please upload an English-language version of your data set.

This will also allow us to determine if your data follows PLOS standards per our Data Availability policy here: https://journals.plos.org/plosone/s/data-availability

8. Please include captions for your Supporting Information files at the end of your manuscript, and update any in-text citations to match accordingly. Please see our Supporting Information guidelines for more information: http://journals.plos.org/plosone/s/supporting-information.

9. If the reviewer comments include a recommendation to cite specific previously published works, please review and evaluate these publications to determine whether they are relevant and should be cited. There is no requirement to cite these works unless the editor has indicated otherwise.

[Note: HTML markup is below. Please do not edit.]

Reviewers' comments:

Reviewer's Responses to Questions

**Comments to the Author**

1. Is the manuscript technically sound, and do the data support the conclusions?

Reviewer #1: Yes

Reviewer #2: Yes

Reviewer #3: Yes

Reviewer #4: Partly

Reviewer #5: Yes

**********

2. Has the statistical analysis been performed appropriately and rigorously?

Reviewer #1: Yes

Reviewer #2: Yes

Reviewer #3: Yes

Reviewer #4: No

Reviewer #5: No

**********

3. Have the authors made all data underlying the findings in their manuscript fully available?

The PLOS Data policy

Reviewer #1: Yes

Reviewer #2: Yes

Reviewer #3: Yes

Reviewer #4: Yes

Reviewer #5: Yes

**********

4. Is the manuscript presented in an intelligible fashion and written in standard English?

Reviewer #1: Yes

Reviewer #2: Yes

Reviewer #3: Yes

Reviewer #4: Yes

Reviewer #5: Yes

**********

Reviewer #1: The paper is generally well written. I have suggested a number of corrections and additions. I ask that you consider them all. The recommendations are explained in detail in the Word document that I have attached to the editor.

Reviewer #2: Cross-sectional and based on self-report data, the study cannot support causal claims. A more appropriate way to present the findings would be in terms of “association” or “indirect association consistent with a mediation model,” rather than effect or prediction. However, the manuscript repeatedly uses causal or intervention-like expressions such as “influence,” “affect,” “alleviate,” and “predict.” The manuscript also presents the curriculum model as if it were an intervention directly producing effects on stress, resilience, and self-efficacy, even though the study is based on a single-time-point questionnaire design. In addition, all participants were students who experienced the same curriculum model, and there is no comparison group. For this reason, the study does not test the actual effect of the curriculum model; it only shows relationships between students’ perceptions of the model and the psychological variables. This point should be stated clearly and consistently throughout the title, abstract, methods, results, discussion, and conclusion.

The manuscript reports that the study started on February 31, 2025. However, the statistical methods section states that all variables were evaluated simultaneously in November 2024. These statements cannot both be correct. The study period, data collection timing, and ethical timeline must be carefully corrected and harmonized throughout the manuscript.

Participants are aged 13–16 years. The manuscript states that informed consent was obtained from all participants, but it does not clearly specify whether parental/guardian consent and/or student assent were also obtained, which is typically required for minors depending on jurisdiction and ethics approval. This should be clarified in detail.

Reviewer #3: The manuscript titled “The relationship between the Chinese physical education and health curriculum model and the psychological stress of junior middle school students: the chain mediating role of psychological resilience and physical self-efficacy” addresses an important and timely topic. The exploration of how structured physical education influences student psychological stress via resilience and self-efficacy is potentially valuable to both educational research and practice. However, several significant issues need to be addressed before the manuscript can be considered for publication.

Major Points:

1.Study Design and Causal Claims

The study employs a cross-sectional design, which limits the ability to draw causal conclusions. While the chain mediation model is statistically appropriate, the discussion frequently implies causality (e.g., statements suggesting the curriculum model“can alleviate psychological stress”). I recommend rephrasing such claims to clearly reflect correlational relationships.

The survey period mentions a non-existent date (February 31, 2025). This should be corrected.

2.Hypothesis Consistency and Interpretation

Hypothesis H1 predicted a negative association between the curriculum model and psychological stress, yet the results indicate a positive relationship. The discussion acknowledges this discrepancy, but a deeper exploration of potential confounding factors, sample characteristics, or alternative explanations is warranted.

The manuscript sometimes conflates direct and indirect effects. While Tables 3 and 4 report these separately, the text should clearly describe the contribution of each pathway in accessible language.

3.Statistical Analysis

Harman’s single-factor test was used to assess common method bias; however, relying solely on this method may be insufficient. Consider complementary approaches or controlling for method variance in structural analyses.

Some regression coefficients and β values appear inconsistent with correlation results. Additionally, confidence intervals for certain indirect effects are reported as negative ranges despite positive effect sizes, which requires clarification.

Providing effect size measures (e.g., Cohen’s d, f²) for key relationships would strengthen interpretability.

4.Writing, Clarity, and Organization

Sentences are often long and complex, which can obscure meaning. Editing for conciseness and readability is strongly recommended.

Figures and tables lack detailed legends, making interpretation difficult without extensive text reference. Ensure all visual elements are self-contained, with clear axis labels and descriptions.

5.Demographic Information

The manuscript provides basic demographic information, but additional details such as socioeconomic status should be included, as these factors can influence psychological stress and resilience.

Minor Points:

Correct minor typographical errors and standardize terminology (e.g., consistently refer to the Chinese physical education and health curriculum model).

Ensure all figures are of sufficient quality for publication, with clearly labeled axes and readable fonts.

Citation formatting should be checked for consistency throughout the manuscript.

Summary Recommendation:

Major Revision. The manuscript has potential to contribute valuable insights to the field, but substantial revisions are needed to address issues in causal interpretation, statistical clarity, and writing quality. Strengthening the discussion of unexpected findings, clarifying mediation pathways, and improving clarity of figures and tables will significantly enhance the manuscript’s scholarly rigor.

Reviewer #4: Whilst the article focuses on an important topic, in its current form it contains serious methodological and interpretative issues. In particular, given the limitations of the research design, it is inappropriate to publish the study without addressing the claims of causality. The authors must undertake comprehensive methodological and conceptual revisions.

Reviewer #5: 1.The time report in the study contains significant inconsistencies. The Methods section states that the research began on "February 31, 2025," a date that does not exist. However, the Statistical Methods section claims that all variables were assessed in "November 2024," creating a temporal contradiction. The authors are requested to verify and unify the data collection timeline, as failure to do so will undermine the study's credibility.

2.Insufficient sample information. The authors should specify the number of schools, regions, and classes involved, the sampling method, inclusion and exclusion criteria, and whether all students received the same course model. The current statement of "nine classes, convenience sampling" is insufficient to assess the representativeness of the sample.

3. Abbreviations of variables in the chart should be annotated upon their first occurrence.

4. There are contradictions in the argumentation. Since the higher the score on the middle school students' psychological stress scale indicates a higher level of psychological stress among them, both the results and discussion sections conclude that the Chinese physical education and health curriculum model positively predicts junior high school students' psychological stress and provide explanations. However, the discussion also mentions that "implementing the Chinese physical education and health curriculum model helps alleviate their psychological stress." The conclusion further states that "the curriculum model can significantly and positively predict junior high school students' psychological stress." This creates a contradiction between the results and the discussion.

Overall evaluation

The manuscript topic has certain research value, and the scales, relevant analyses, and mediation models employed are largely consistent with existing research directions in physical activity, psychological resilience, self-efficacy, and stress. Existing studies also support the association or mediating relationships between physical activity and stress, psychological resilience, and self-efficacy in adolescents. However, the current manuscript has several critical issues that affect the accurate reporting of results and scientific validity, and major revisions are recommended before further consideration.

**********

what does this mean?). If published, this will include your full peer review and any attached files.

If you choose “no”, your identity will remain anonymous but your review may still be made public.

**Do you want your identity to be public for this peer review?**  For information about this choice, including consent withdrawal, please see our Privacy Policy

Reviewer #1: **Yes:**  Professor Dušan Perić, PhD

Reviewer #2: No

Reviewer #3: No

Reviewer #4: No

Reviewer #5: No

**********

[NOTE: If reviewer comments were submitted as an attachment file, they will be attached to this email and accessible via the submission site. Please log into your account, locate the manuscript record, and check for the action link "View Attachments". If this link does not appear, there are no attachment files.]

To ensure your figures meet our technical requirements, please review our figure guidelines: https://journals.plos.org/plosone/s/figures

You may also use PLOS’s free figure tool, NAAS, to help you prepare publication quality figures: https://journals.plos.org/plosone/s/figures#loc-tools-for-figure-preparation.

NAAS will assess whether your figures meet our technical requirements by comparing each figure against our figure specifications.

Attachment Submitted filename: Observations & Suggestions.docx

Attachment Submitted filename: review.docx

Attachment Submitted filename: REVIEWER.docx

# Author comment: Author response to Decision Letter 1

**DOI:** 10.1371/journal.pone.0360804.r002

25 Jun 2026

All comments raised by the reviewers have been fully addressed one by one. Detailed point-by-point responses are provided in the attached response letter, and all corresponding revisions have been highlighted in the revised manuscript.

Attachment Submitted filename: Response to Reviewer 5.docx

# Editor report: Decision Letter 1

**DOI:** 10.1371/journal.pone.0360804.r003

**Authors:** Jiajia Ye

12 Jul 2026

Please submit your revised manuscript by Aug 26 2026 11:59PM. If you will need more time than this to complete your revisions, please reply to this message or contact the journal office at plosone@plos.org. When you're ready to submit your revision, log on to https://www.editorialmanager.com/pone/ and select the 'Submissions Needing Revision' folder to locate your manuscript file.

- A letter that responds to each point raised by the academic editor and reviewer(s). You should upload this letter as a separate file labeled 'Response to Reviewers'.
- A marked-up copy of your manuscript that highlights changes made to the original version. You should upload this as a separate file labeled 'Revised Manuscript with Track Changes'.
- An unmarked version of your revised paper without tracked changes. You should upload this as a separate file labeled 'Manuscript'.

If you would like to make changes to your financial disclosure, please include your updated statement in your cover letter. Guidelines for resubmitting your figure files are available below the reviewer comments at the end of this letter.

If applicable, we recommend that you deposit your laboratory protocols in protocols.io to enhance the reproducibility of your results. Protocols.io assigns your protocol its own identifier (DOI) so that it can be cited independently in the future. For instructions see: https://journals.plos.org/plosone/s/submission-guidelines#loc-laboratory-protocols. Additionally, PLOS ONE offers an option for publishing peer-reviewed Lab Protocol articles, which describe protocols hosted on protocols.io. Read more information on sharing protocols at https://plos.org/protocols?utm_medium=editorial-email&utm_source=authorletters&utm_campaign=protocols.

As the corresponding author, your ORCID iD is verified in the submission system and will appear in the published article. PLOS supports the use of ORCID, and we encourage all coauthors to register for an ORCID iD and use it as well. Please encourage your coauthors to verify their ORCID iD within the submission system before final acceptance, as unverified ORCID iDs will not appear in the published article. *Only*  the individual author can complete the verification step; PLOS staff *cannot*  verify ORCID iDs on behalf of authors.

We look forward to receiving your revised manuscript.

Kind regards,

Jiajia Ye

Guest Editor

PLOS One

Journal Requirements:

If the reviewer comments include a recommendation to cite specific previously published works, please review and evaluate these publications to determine whether they are relevant and should be cited. There is no requirement to cite these works unless the editor has indicated otherwise.

[Note: HTML markup is below. Please do not edit.]

[NOTE: If reviewer comments were submitted as an attachment file, they will be attached to this email and accessible via the submission site. Please log into your account, locate the manuscript record, and check for the action link "View Attachments". If this link does not appear, there are no attachment files.]

To ensure your figures meet our technical requirements, please review our figure guidelines: https://journals.plos.org/plosone/s/figures

You may also use PLOS’s free figure tool, NAAS, to help you prepare publication quality figures: https://journals.plos.org/plosone/s/figures#loc-tools-for-figure-preparation.

NAAS will assess whether your figures meet our technical requirements by comparing each figure against our figure specifications.

# Author comment: Author response to Decision Letter 2

**DOI:** 10.1371/journal.pone.0360804.r004

18 Jul 2026

All required revisions have been fully finished. All adjusted contents are marked in the tracked manuscript, and detailed point-by-point replies to every comment are provided in the separate Response to Reviewers file.

Attachment Submitted filename: Response to Editorial Comments.docx

# Peer review: Decision Letter 2

**DOI:** 10.1371/journal.pone.0360804.r005

**Authors:** Jiajia Ye

1 Sep 2026

Dear Dr. Ma，

Thank you for submitting your manuscript to PLOS One. After careful consideration, we feel that it has merit but does not fully meet PLOS One’s publication criteria as it currently stands. Therefore, we invite you to submit a revised version of the manuscript that addresses the points raised during the review process.

Please submit your revised manuscript by Oct 16 2026 11:59PM. If you will need more time than this to complete your revisions, please reply to this message or contact the journal office at plosone@plos.org. When you're ready to submit your revision, log on to https://www.editorialmanager.com/pone/ and select the 'Submissions Needing Revision' folder to locate your manuscript file.

- A letter that responds to each point raised by the academic editor and reviewer(s). You should upload this letter as a separate file labeled 'Response to Reviewers'.
- A marked-up copy of your manuscript that highlights changes made to the original version. You should upload this as a separate file labeled 'Revised Manuscript with Track Changes'.
- An unmarked version of your revised paper without tracked changes. You should upload this as a separate file labeled 'Manuscript'.

If you would like to make changes to your financial disclosure, please include your updated statement in your cover letter. Guidelines for resubmitting your figure files are available below the reviewer comments at the end of this letter.

If applicable, we recommend that you deposit your laboratory protocols in protocols.io to enhance the reproducibility of your results. Protocols.io assigns your protocol its own identifier (DOI) so that it can be cited independently in the future. For instructions see: https://journals.plos.org/plosone/s/submission-guidelines#loc-laboratory-protocols. Additionally, PLOS One offers an option for publishing peer-reviewed Lab Protocol articles, which describe protocols hosted on protocols.io. Read more information on sharing protocols at https://plos.org/protocols?utm_medium=editorial-email&utm_source=authorletters&utm_campaign=protocols.

As the corresponding author, your ORCID iD is verified in the submission system and will appear in the published article. PLOS supports the use of ORCID, and we encourage all coauthors to register for an ORCID iD and use it as well. Please encourage your coauthors to verify their ORCID iD within the submission system before final acceptance, as unverified ORCID iDs will not appear in the published article. *Only*  the individual author can complete the verification step; PLOS staff *cannot*  verify ORCID iDs on behalf of authors.

We look forward to receiving your revised manuscript.

Kind regards,

Jiajia Ye

Guest Editor

PLOS One

Journal Requirements:

If the reviewer comments include a recommendation to cite specific previously published works, please review and evaluate these publications to determine whether they are relevant and should be cited. There is no requirement to cite these works unless the editor has indicated otherwise.

Please review your reference list to ensure that it is complete and correct. If you have cited papers that have been retracted, please include the rationale for doing so in the manuscript text, or remove these references and replace them with relevant current references. Any changes to the reference list should be mentioned in the rebuttal letter that accompanies your revised manuscript. If you need to cite a retracted article, indicate the article’s retracted status in the References list and also include a citation and full reference for the retraction notice.

[Note: HTML markup is below. Please do not edit.]

Reviewers' comments:

Reviewer's Responses to Questions

**Comments to the Author**

Reviewer #1: (No Response)

Reviewer #2: (No Response)

Reviewer #3: All comments have been addressed

**********

2. Is the manuscript technically sound, and do the data support the conclusions?

Reviewer #1: Yes

Reviewer #2: Partly

Reviewer #3: Yes

**********

3. Has the statistical analysis been performed appropriately and rigorously?

Reviewer #1: Yes

Reviewer #2: Yes

Reviewer #3: Yes

**********

4. Have the authors made all data underlying the findings in their manuscript fully available?

The PLOS Data policy

Reviewer #1: Yes

Reviewer #2: Yes

Reviewer #3: Yes

**********

5. Is the manuscript presented in an intelligible fashion and written in standard English?

Reviewer #1: Yes

Reviewer #2: Yes

Reviewer #3: Yes

**********

Reviewer #1: I suggest that you keep the full name of the curriculum ("Chinese physical education and health curriculum model") only in the title, and use the abbreviation CPEH throughout the text (which you did). Based just on the title, It will be unclear to an uninformed reader looking for relevant references what CPEH is. You have taken all my previous recommendations into account and I think the paper is now ready for publication. Compliments for your effort.

Reviewer #2: The title still begins with “Effects of the CPEH Curriculum model...,” which implies that the study evaluated the effects of the curriculum.

In the Abstract, the authors refer to “four influencing pathways” and the “direct effect of the curriculum model.”

Hypothesis 1 states that the curriculum “directly and negatively predicts psychological stress.”

Hypothesis 3 similarly states that physical self-efficacy “negatively predicts psychological stress.”

The caption of Figure 1 refers to a model of the “influence” of the CPEH Curriculum.

The Results section repeatedly uses the expression “significant positive predictive effect.”

The Conclusion states that “the CPEH Curriculum can positively predict students’ psychological stress.”

Because all variables were measured simultaneously and there was no control or comparison group, these expressions remain stronger than the study design permits. I therefore recommend replacing them consistently with terms such as “association,” “relationship,” “was associated with,” or “indirect association consistent with the proposed mediation model.” In particular, the title should be revised to reflect the relational nature of the study.

Reviewer #3: Overall Assessment

The authors have made substantial revisions in response to the comments raised during the previous review rounds. The revised manuscript demonstrates clear improvement in several important aspects, particularly regarding the interpretation of the cross-sectional study design, clarification of study limitations, reduction of potentially causal language, and consideration of alternative explanations for unexpected findings.

A particularly important improvement is the revised interpretation of the relationship between the CPEH Curriculum and psychological stress. The authors have appropriately recognized that the current study is based on cross-sectional questionnaire data and therefore cannot establish causal relationships. The revised manuscript consistently emphasizes that the identified pathways represent statistical associations rather than confirmed causal mechanisms. This revision substantially improves the scientific rigor of the manuscript and appropriately aligns the conclusions with the study design.

Overall, the manuscript has been significantly strengthened and the remaining issues are primarily related to conceptual consistency, reporting transparency, and presentation quality. Addressing the following minor comments would further improve the clarity and reproducibility of the study before final consideration for publication in PLOS ONE.

Minor Comments

1. Clarification and consistency of terminology and abbreviations

The manuscript uses several abbreviations and measurement-related terms, including CPEH, PSES, and psychological resilience-related measures. Although these abbreviations are appropriately defined when first introduced, the authors are encouraged to conduct a final review to ensure consistent usage throughout the entire manuscript, including the abstract, main text, tables, and figures.

In addition, some overlapping terms, such as “psychological stress,” “stress level,” and “mental stress,” appear to be used interchangeably. These terms may imply different conceptual meanings in psychological research. Therefore, the authors should clarify whether these terms refer to the same underlying construct or represent distinct dimensions. If they refer to the same construct, a unified term should be adopted throughout the manuscript. If differences exist, the conceptual distinctions should be explicitly explained.

Improving terminology consistency would enhance conceptual precision and reduce potential ambiguity for readers.

2. Further description of the CPEH Curriculum implementation

The CPEH Curriculum remains a central component of the current study; however, the description of its implementation is still relatively limited. Although the revised manuscript provides additional information, more contextual details would improve the transparency and interpretability of the findings.

The authors are encouraged to further describe:

• the specific components and educational objectives of the CPEH Curriculum;

• the duration, frequency, and overall implementation process;

• teacher qualifications, training procedures, or requirements for curriculum delivery;

• the major differences between the CPEH Curriculum and conventional physical education classes.

Providing additional details would help readers from different educational contexts better understand the intervention characteristics and evaluate the potential applicability and reproducibility of the findings.

3. Improvement of tables and figures presentation

The tables and figures are generally informative and contribute effectively to the presentation of the results. However, several minor improvements are recommended to enhance clarity and ensure accurate interpretation.

The authors should ensure that:

• all abbreviations used in tables are clearly defined in table notes;

• decimal places are standardized throughout all statistical results;

• figure legends provide sufficient explanation so that figures can be understood independently from the main text.

For the mediation model figure, the authors should explicitly indicate whether the reported path coefficients represent standardized or unstandardized estimates. This information is important for readers to correctly interpret the magnitude and meaning of the reported effects.

**********

what does this mean?). If published, this will include your full peer review and any attached files.

If you choose “no”, your identity will remain anonymous but your review may still be made public.

**Do you want your identity to be public for this peer review?**  For information about this choice, including consent withdrawal, please see our Privacy Policy

Reviewer #1: **Yes:**  Professor Dušan Perić, PhD (Academy of applied studies Šabac)

Reviewer #2: No

Reviewer #3: No

**********

[NOTE: If reviewer comments were submitted as an attachment file, they will be attached to this email and accessible via the submission site. Please log into your account, locate the manuscript record, and check for the action link "View Attachments". If this link does not appear, there are no attachment files.]

To ensure your figures meet our technical requirements, please review our figure guidelines: https://journals.plos.org/plosone/s/figures

You may also use PLOS’s free figure tool, NAAS, to help you prepare publication quality figures: https://journals.plos.org/plosone/s/figures#loc-tools-for-figure-preparation.

NAAS will assess whether your figures meet our technical requirements by comparing each figure against our figure specifications.

# Author comment: Author response to Decision Letter 3

**DOI:** 10.1371/journal.pone.0360804.r006

2 Sep 2026

Dear Editor,

We would like to submit the revised manuscript entitled Associations between the Chinese physical education and health curriculum model and psychological stress among junior middle school students: the chain-mediating role of psychological resilience and physical self-efficacy for reconsideration for publication.

We sincerely appreciate you and the reviewers for your careful evaluation and constructive comments, which have greatly helped us improve the quality and rigor of this manuscript. We have carefully addressed every comment raised by Reviewer 1, Reviewer 2 and Reviewer 3. All revisions have been implemented in the revised manuscript, and modifications are highlighted with yellow colour within the text. A point-by-point response to all reviewer comments is attached below.

Major revisions performed in this version include:

1. Removal of causal-oriented wording: The manuscript title was revised from “Effects of …” to “Associations between …”. We have systematically eliminated causal-interpretation wording across the title, abstract, research hypotheses, figure captions, results and conclusion sections. Explicit statements are added to emphasise that cross-sectional data cannot support causal inferences.

2. Unification of abbreviations and terminology: We completed a full-manuscript check to standardise all abbreviations and variable terms. Redundant overlapping expressions for stress‑related constructs were consolidated.

3. Supplement of intervention details: A new subsection CPEH Curriculum Implementation was added within the Methods section, elaborating curriculum components, educational objectives, implementation procedures, teacher training requirements, and differences relative to conventional physical education classes.

4. Standardisation of tables and figures: Abbreviations in table notes are fully defined; decimal places for all statistical results are unified to two decimal places; figure legends are enriched for self-contained readability; and mediation-model legends explicitly note that path coefficients are standardized estimates.

All original statistical outputs (β values, t-values, p-values, Bootstrap confidence intervals) remain unchanged; only descriptive and interpretive texts have been modified.

We believe the manuscript has been substantially improved and meets the journal’s publication requirements. Thank you again for your time and consideration. We look forward to your favourable reply.

Sincerely,

Corresponding Author: Aimin Ma

Nantong University, Nantong, Jiangsu Province, 226019, China

Email: 50994546@qq.com

Attachment Submitted filename: Response to Reviewers 3.docx

# Peer review: Decision Letter 3

**DOI:** 10.1371/journal.pone.0360804.r007

**Authors:** Jiajia Ye

1 Oct 2026

Associations between the Chinese physical education and healthCurriculum model to the psychological stress of junior middle school students: the chain mediating role of psychological resilienceand physical self-efficacy

PONE-D-25-61805R3

Dear Dr. Ma,

We’re pleased to inform you that your manuscript has been judged scientifically suitable for publication and will be formally accepted for publication once it meets all outstanding technical requirements.

Within one week, you’ll receive an e-mail detailing the required amendments. When these have been addressed, you’ll receive a formal acceptance letter and your manuscript will be scheduled for publication.

An invoice will be generated when your article is formally accepted. Please note, if your institution has a publishing partnership with PLOS and your article meets the relevant criteria, all or part of your publication costs will be covered. Please make sure your user information is up-to-date by logging into Editorial Manager at Editorial Manager® and clicking the ‘Update My Information' link at the top of the page. For questions related to billing, please contact billing support.

If your institution or institutions have a press office, please notify them about your upcoming paper to help maximize its impact. If they’ll be preparing press materials, please inform our press team as soon as possible -- no later than 48 hours after receiving the formal acceptance. Your manuscript will remain under strict press embargo until 2 pm Eastern Time on the date of publication. For more information, please contact onepress@plos.org.

Kind regards,

Jiajia Ye

Guest Editor

PLOS One

Additional Editor Comments (optional):

Reviewers' comments:

Reviewer's Responses to Questions

**Comments to the Author**

Reviewer #2: All comments have been addressed

**********

2. Is the manuscript technically sound, and do the data support the conclusions?

Reviewer #2: Partly

**********

3. Has the statistical analysis been performed appropriately and rigorously?

Reviewer #2: Yes

**********

4. Have the authors made all data underlying the findings in their manuscript fully available?

The PLOS Data policy

Reviewer #2: Yes

**********

5. Is the manuscript presented in an intelligible fashion and written in standard English?

Reviewer #2: Yes

**********

Reviewer #2: The revisions are sufficient in their current form. The authors have adequately addressed the comments and concerns raised during the review process. The manuscript has been improved accordingly, and I believe that no further major revisions are necessary. In its revised form, the manuscript is suitable for publication and can be accepted.

**********

what does this mean?). If published, this will include your full peer review and any attached files.

If you choose “no”, your identity will remain anonymous but your review may still be made public.

**Do you want your identity to be public for this peer review?**  For information about this choice, including consent withdrawal, please see our Privacy Policy

Reviewer #2: No

**********

# Editor report: Acceptance letter

**DOI:** 10.1371/journal.pone.0360804.r008

**Authors:** Jiajia Ye

PONE-D-25-61805R3

PLOS One

Dear Dr. Ma,

I'm pleased to inform you that your manuscript has been deemed suitable for publication in PLOS One. Congratulations! Your manuscript is now being handed over to our production team.

At this stage, our production department will prepare your paper for publication. This includes ensuring the following:

* All references, tables, and figures are properly cited

* All relevant supporting information is included in the manuscript submission,

* There are no issues that prevent the paper from being properly typeset

You will receive further instructions from the production team, including instructions on how to review your proof when it is ready. Please keep in mind that we are working through a large volume of accepted articles, so please give us a few days to review your paper and let you know the next and final steps.

Lastly, if your institution or institutions have a press office, please let them know about your upcoming paper now to help maximize its impact. If they'll be preparing press materials, please inform our press team within the next 48 hours. Your manuscript will remain under strict press embargo until 2 pm Eastern Time on the date of publication. For more information, please contact onepress@plos.org.

You will receive an invoice from PLOS for your publication fee after your manuscript has reached the completed accept phase. If you receive an email requesting payment before acceptance or for any other service, this may be a phishing scheme. Learn how to identify phishing emails and protect your accounts at https://explore.plos.org/phishing.

If we can help with anything else, please email us at customercare@plos.org.

Thank you for submitting your work to PLOS One and supporting open access.

Kind regards,

PLOS One Editorial Office Staff

on behalf of

Dr. Jiajia Ye

Guest Editor

PLOS One
